# BEExact: a Metataxonomic Database Tool for High-Resolution Inference of Bee-Associated Microbial Communities

**DOI:** 10.1128/mSystems.00082-21

**Published:** 2021-04-06

**Authors:** Brendan A. Daisley, Gregor Reid

**Affiliations:** a Department of Microbiology & Immunology, The University of Western Ontario, London, Ontario, Canada; b Canadian Centre for Human Microbiome and Probiotics Research, London, Ontario, Canada; c Department of Surgery, Schulich School of Medicine, London, Ontario, Canada; University of Waterloo

**Keywords:** microbiota, bees, 16S rRNA gene sequencing, microbial ecology, bioinformatics, host-microbe interactions, polymicrobial communities, microbial phylogenetics, endosymbionts, environmental microbiology, invertebrate-microbe interactions, microbial communities, taxonomy, metataxonomics

## Abstract

High-throughput 16S rRNA gene sequencing technologies have robust potential to improve our understanding of bee (Hymenoptera: Apoidea)-associated microbial communities and their impact on hive health and disease. Despite recent computation algorithms now permitting exact inferencing of high-resolution exact amplicon sequence variants (ASVs), the taxonomic classification of these ASVs remains a challenge due to inadequate reference databases. To address this, we assemble a comprehensive data set of all publicly available bee-associated 16S rRNA gene sequences, systematically annotate poorly resolved identities via inclusion of 618 placeholder labels for uncultivated microbial dark matter, and correct for phylogenetic inconsistencies using a complementary set of distance-based and maximum likelihood correction strategies. To benchmark the resultant database (BEExact), we compare performance against all existing reference databases *in silico* using a variety of classifier algorithms to produce probabilistic confidence scores. We also validate realistic classification rates on an independent set of ∼234 million short-read sequences derived from 32 studies encompassing 50 different bee types (36 eusocial and 14 solitary). Species-level classification rates on short-read ASVs range from 80 to 90% using BEExact (with ∼20% due to “bxid” placeholder names), whereas only ∼30% at best can be resolved with current universal databases. A series of data-driven recommendations are developed for future studies. We conclude that BEExact (https://github.com/bdaisley/BEExact) enables accurate and standardized microbiota profiling across a broad range of bee species—two factors of key importance to reproducibility and meaningful knowledge exchange within the scientific community that together, can enhance the overall utility and ecological relevance of routine 16S rRNA gene-based sequencing endeavors.

**IMPORTANCE** The failure of current universal taxonomic databases to support the rapidly expanding field of bee microbiota research has led to many investigators relying on “in-house” reference sets or manual classification of sequence reads (usually based on BLAST searches), often with vague identity thresholds and subjective taxonomy choices. This time-consuming, error- and bias-prone process lacks standardization, cripples the potential for comparative cross-study analysis, and in many cases is likely to incorrectly sway study conclusions. BEExact is structured on and leverages several complementary bioinformatic techniques to enable refined inference of bee host-associated microbial communities without any other methodological modifications necessary. It also bridges the gap between current practical outcomes (i.e., phylotype-to-genus level constraints with 97% operational taxonomic units [OTUs]) and the theoretical resolution (i.e., species-to-strain level classification with 100% ASVs) attainable in future microbiota investigations. Other niche habitats could also likely benefit from customized database curation via implementation of the novel approaches introduced in this study.

## INTRODUCTION

Next generation sequencing (NGS) technologies are heavily utilized for characterizing microbial communities. They can provide insight into the biological relevance of interacting species as well as their ecological functions in a given ecosystem. Subgenus-level identification of taxa is considered most valuable in gaining a deeper functional understanding of host-associated microbial community dynamics, as many ecologically important traits are specific for species to strains (1). However, accurate microbial identification at high resolution (i.e., low taxonomic rank) remains a challenge when studying many niche environments due to the lack of high-identity taxonomic references in publicly available “universal” databases.

For over a decade now, molecular biology-based profiling of bee (Hymenoptera: family Apidae)-associated microbial communities has been a major global interest in efforts to control the spread of infectious diseases and reduce population decline of these important pollinators (2). Largely stemming from the desire to classify novel or unannotated sequences into processable and comparable taxonomic groupings without prior information of reference taxonomy, most published literature thus far has used clustering algorithms to group similar sequences (usually at 97% identity) into operational taxonomic units (OTUs) (3). Consequently, this approach constrains taxonomic resolution to the genus level since sequence matching at 99 to 100% identity is the only appropriate method for species- to strain-level assignment of 16S amplicon data (4)—though this is not an intrinsic limitation to 16S rRNA gene sequencing technologies as a whole. Many newer denoising algorithms (e.g., DADA2, Unoise3, Deblur [5], ampliCI [6]) that do not depend on similarity thresholds can parse sequence reads into exact amplicon sequence variants (ESVs/ASVs; synonymous high-resolution analogues of the traditional OTU) that can detect single-nucleotide polymorphisms and allow species- to strain-level assignment of reads (7). The realization of amplicon sequence variant superiority in terms of precise microbial identification has led to this approach being implemented in several large-scale initiatives, including the American Gut Project and the Earth Microbiome Project (8).

Nonetheless, the potential of ASVs is often limited by sequence length, information density of the specific hypervariable region(s) targeted, and especially the availability of well-characterized reference databases for classification of reads. Recent advancements in high-throughput sequencing instruments (e.g., PacBio, Oxford Nanopore, and Illumina shotgun metagenomic sequencing) have made nearly full-length 16S rRNA gene sequencing possible, which addresses sequence length concerns. However, while these methods hold great promise for the future of microbial ecology, they do not solve the issue of missing or poorly characterized reference sequences and their prohibitive costs restrict feasibility in population-level or other large-scale studies. Moreover, the taxonomic resolution achievable from sequencing of any given 16S rRNA gene region is highly habitat specific (9). For example, in comparison to the commonly sequenced V3-V4 region, the V1-V3 region was recently shown to be more effective for distinguishing taxa at the species level in the human aerodigestive tract (9). It is therefore critical to assess which 16S rRNA gene region(s) provides the most informative representation of taxa associated with the specific environment being studied.

The importance of a comprehensive reference database and habitat-directed 16S rRNA gene region selection is particularly relevant to closely related hosts, such as bee species within the superfamily Apoidea. Corbiculate bees (subfamily Apinae, clade Anthophila) likely provide the best example, given the consistency in observing a similar set of core microbes across different lineages independent of geography or sympatry (10). Many of these core microbiota members, such as various *Gilliamella* spp. for example (originally grouped within the “Gamma-1” phylotype clustered at 97% identity [11]), have since been validly published as separate species and received their own names with Standing in Nomenclature as per the International Code of Nomenclature of Prokaryotes (12). This improved resolution has also revealed that many species are closely related anatomical site specialists that share local resources but perform differential roles within distinct niche communities along the intestinal tract (13). Thus, being able to accurately distinguish between closely related species would add considerable value to routine 16S rRNA gene sequencing studies. Corroborating this, a recent honey bee metagenomic survey has pronounced the need to move beyond the long time standard of phylotype-level microbiota characterization (14). While efforts have been initiated toward the development of dedicated data portals, like BeeBiome (15), to date, these resources primarily support whole-genome sequencing and were last updated in 2016. Due to the overall lack of available resources at large, bee researchers frequently rely on universal databases (e.g., SILVA, RDP, GreenGenes) to achieve taxonomic classification of 16S rRNA gene sequencing data.

These large all-purpose databases contain an expansive set of phylogenetically diverse reference sequences that are broadly applicable to a variety of habitats. In most cases, this generality improves workflow simplicity and provides reasonable estimation of taxonomy down to the genus level. In contrast, they lack comprehensiveness in habitat-specific taxonomic references and do not encompass the full range of sequence representatives expected to be found in any one habitat. Moreover, annotation error rates can reach near 20% using these databases due to the inclusion of misannotated 16S rRNA gene sequences and revision lag in adapting the most up-to-date taxonomic naming conventions (16). Current approaches to overcoming this include constructing habitat-specific databases by either (i) generating novel references using long-read sequencing technologies (e.g., RIM-DP for rumen [17], HITdb for human colon [18], and eHOMD for human aerodigestive tract [1]) or (ii) compiling a curated list of representatives already available in public data repositories (e.g., DictDB for termites [19], MiDAS 2.0 for biological wastewater treatment systems [20], DAIRYdb for dairy products [21], FreshTrain+TaxAss for freshwater fish [22], and HBDB for honey bees [23]). The latter database, HBDB, is largely outdated but was fundamental in early microbiota studies on Apis mellifera by significantly reducing misclassification error rates and allowing phylotype (assigned at the family-level) taxonomic resolution.

Another important aspect of assigning taxonomy to sequence reads is the classifier used, which can impact overall consistency and accuracy of classifications irrespective to that of the taxonomic references provided. Current 16S pipelines like mothur (24), KRAKEN2 (25), DECIPHER (26), DADA2 (27), and QIIME2 (28) implement a variety of classifiers. Notably, the naïve Bayesian classifier (29) is the one most commonly used due to availability of frequently updated universal taxonomy databases formatted for its use, its computational efficiency, and its adaptability for improving classification rates (29–31). The latter is potentiated through supervised learning (i.e., machine learning that maps an input to an output, based on inference from input-output training data) for which unambiguous classification of sequences is conditionally dependent on the occurrence and abundance of differentiating examples provided in the reference training set. According to these stipulations, supplying a comprehensive and accurately annotated reference training set tailored to a specific environment is expected to greatly enhance confidence, accuracy, and depth of classification for sequences found in the same or similar environments. Recently developed classifier algorithms like SINTAX (32) and IDTAXA (33) also provide similar performances but report reduced error rates compared to standards set by the naïve Bayesian classifier (29). Importantly, despite the algorithm used, classification rates are restricted by the accuracy and completeness of the applied reference sequences used in training steps.

There continues to be persistent biotic and abiotic threats to bee species, which are major pollinators for the world’s food supply. Thus, it is critical to understand how associated microbial communities modulate resistance to these stressors. Currently, bee microbiota investigations suffer from inconsistent use of classification methods, unclear 16S rRNA gene region selection, and jejune representation of habitat-specific references in commonly applied universal training databases. To address these issues, in this study, the goals were as follows: (i) to identify the most informative 16S rRNA gene region for profiling bee-associated microbial communities as a selective guide for future studies, (ii) to develop a comprehensively annotated reference sequence database (BEExact) for high-precision assignment of taxonomy to high-resolution ASVs, (iii) to benchmark the developed database against existing universal databases using a variety of taxonomic classifier algorithms, and (iv) to validate realistic classification performance on available 16S rRNA gene sequencing data sets from past bee microbiota studies.

## RESULTS

### BEExact database construction and curation.

**FIG 1:**
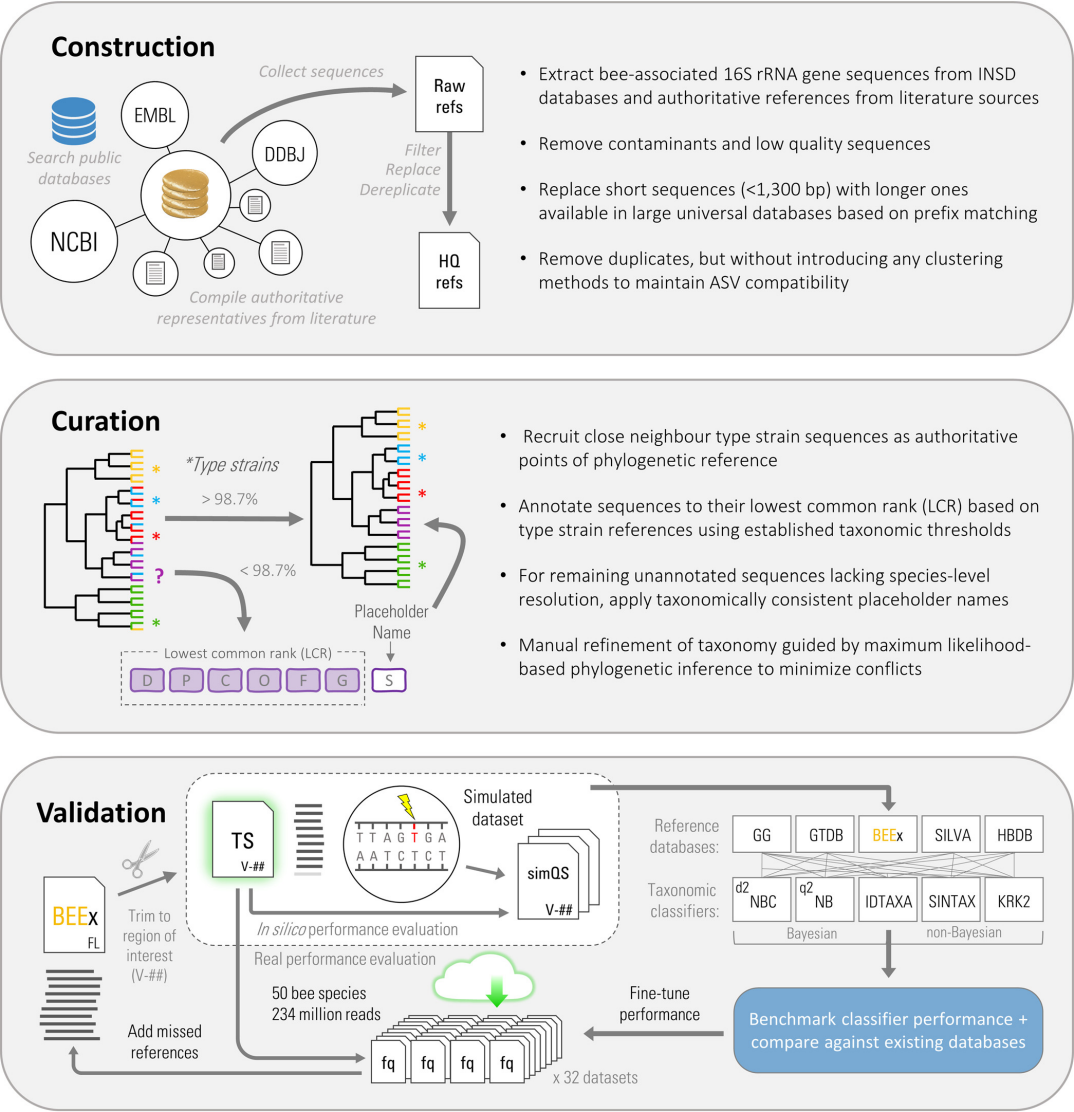
Overview of study design. Briefly, the initial BEExact database was generated by collecting a set of all bee-associated 16S rRNA gene sequences available from public databases or literature sources. The sequences were then extensively curated to correct for mislabeled taxonomic representatives as well as to provide placeholder names to uncultured microbial dark matter. The performance of classifier algorithms was compared *in silico* to determine the optimal choice, followed by comparisons to existing databases. In a final validation step, BEExact was tested on 32 data sets to demonstrate its capacity to enable confident classification of bee host-associated microbial communities. Sequence representatives from missed taxa were supplemented to the final database to maximize comprehension.

A schematic overview of the study design is provided in Fig. 1. Initial construction of BEExact was performed by searching for bacterial 16S rRNA gene sequences available from the International Nucleotide Sequence Databases (INSD) (including NCBI, EMBL, and DDBJ) using all known bee families within the clade Anthophila as keywords (e.g., “Apidae,” “Megachilidae,” “Stenotritidae,” etc.) as well as respective common names when applicable. Additional sequence representatives were also collected from relevant literature sources (34–48). This initial compilation step captured 8,869 total sequence representatives with the top 10 bee hosts (per genus by the number of associated 16S rRNA gene sequences) being *Apis* (4,106), *Bombus* (637), *Hesperapis* (349), *Diadasia* (347), *Megachile* (338), *Redviva* (333), *Halictus* (305), *Xylocopa* (305), *Colletes* (301), and *Calliopsis* (282). Lower-quality sequences were filtered out based on sequence length (<1,300 bp) and replaced, if possible, with higher-quality representatives (>99% percent identity) from the latest SILVA v138, GreenGenes v13.8, RDP v18, and GTDB r95 databases. After removal of duplicates, chimeras, sequences with suspiciously long V4 regions, and contaminating sequences of nonbacterial origin, the intermediate BEExact database contained 4,518 bee host-associated 16S rRNA gene sequence representatives. The preprocessed redundant accession list containing the original 8,869 sequences (see Data Set S1A in the supplemental material) and the mapping file to the nonredundant 4,518 quality-filtered identifiers (Data Set S1B) are provided for completeness and traceback inquiries.

10.1128/mSystems.00082-21.9 DATA SET S1 Database sequence mapping files, primer sequences, primer biases, simulated error results, cross validation results, nonredundant ASV list, and empirical classification results. Download Data Set S1, XLSX file, 32.9 MB. Copyright © 2021 Daisley and Reid. 2021 Daisley and Reid. https://creativecommons.org/licenses/by/4.0/ This content is distributed under the terms of the Creative Commons Attribution 4.0 International license.

Strictly based on designations of taxonomy identifiers (NCBI:txid numbers) associated with each accession, only 1,620 sequences (35.9%) were initially annotated at the species level—likely representing an artifact of either lacking reference taxonomy at the time of sequence submission, misannotated environmental sequences, or public database inconsistencies preventing consensus labels. To improve taxonomic resolution in the data set, unannotated sequences were queried against type strain material (at >98.7% similarity based on species-level cutoff [49]) in GenBank as well as the latest reference (i.e., nonclustered) versions of SILVA and RDP. This step successfully increased total annotations at the species level to 3853 (85.3%). The remaining dark matter sequences lacking adequate similarity to be assigned taxonomy at the species level were instead annotated down to their lowest common rank (LCR) based on established thresholds (49, 50). Subsequently, we implemented a novel method of *de novo* taxonomy approximation (see Materials and Methods for details) to generate phylogenetically consistent placeholder names and achieve complete taxonomic lineage integrity for all sequences in the BEExact database (Table 1).

**TABLE 1:** Number of placeholder names for unculturable (or yet to be cultured) taxa following phylogenetic correction to distance-based group memberships at each taxonomic rank

Taxonomic rank	No. of valid species names^*a*^	No. of *de novo* placeholder (bxid) names^*b*^
Phylum	4,518	0
Class	4,518	0
Order	4,514	4
Family	4,509	9
Genus	4,437	81
Species	3,900	618

a Sequences with species-level annotations based on >98.7% identity with type strain representatives.

b Placeholder names given to sequences with less than <98.7% identity with type strain representatives.

As an additional form of quality assurance, manual inspection of taxonomy was performed as previously described (16) by correcting taxonomic inconsistencies in which members of the same taxonomic rank were present with dissimilar taxonomic lineages due to mislabeling or outdated naming conventions. Furthermore, we recruited a set of close neighbor (CN) type strain sequences as authoritative points of reference which were used with an established semiautomated phylogeny-aware taxonomy improvement and validation algorithm (51) to correct for branching errors in monophyletic taxonomic groups. Altogether, these curations steps enabled a stable taxonomic reference point to be developed for all sequence representatives and greatly improved overall robustness and accuracy.

The BEExact reference data set (*BEEx-FL-refs*) that was used for all subsequent benchmarking and validation experiments contains 4,518 nearly full-length bee host-associated bacterial 16S rRNA gene sequences consisting of 11 phyla, 17 classes, 57 orders, 96 families, 219 genera, and 643 species (Data Set S1C). *Gammaproteobacteria* (52.9%), *Bacilli* (25.2%), *Alphaproteobacteria* (11.6%), and *Actinobacteria* (7.8%) dominated the database at the class level. Additionally, enrichment in many of the species that make up the core microbiota of eusocial corbiculate bees (52), including *Gilliamella*, *Snodgrassella*, *Lactobacillus*, *Apilactobacillus*, and *Bombilactobacillus* spp., was observed.

### Evaluation of primer sets used for 16S rRNA gene sequencing.

**FIG 2:**
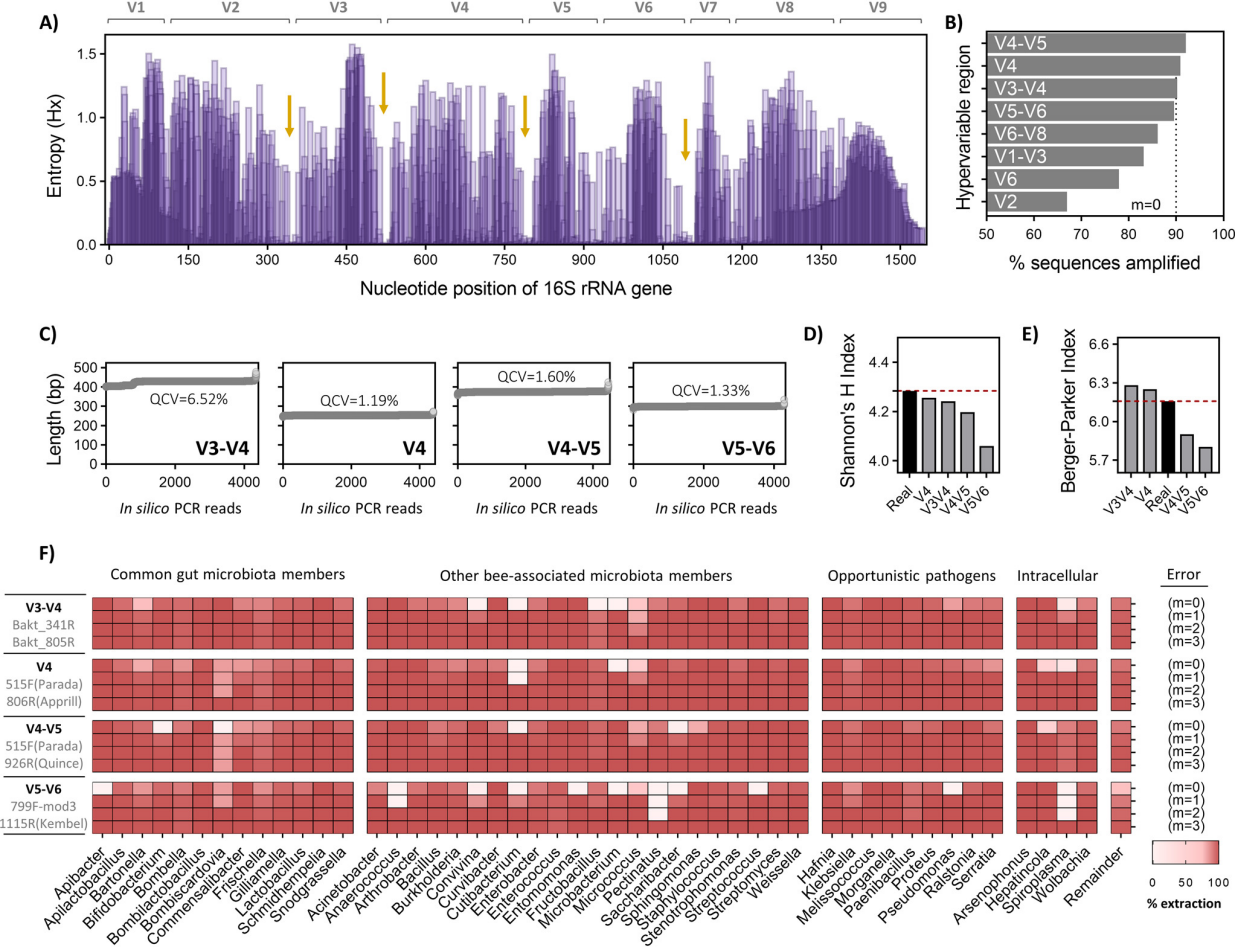
Comparison of hypervariable regions and common primer sets for classification of honey bee-specific taxa encompassed within the BEExact database. (A) Shannon’s entropy was calculated for each position on a 50,000 bp alignment of the 16S rRNA gene. Data shown as de-gapped representative sequences of equal length matching aligned E. coli 16S rRNA gene positions 30-1390. Orange arrows indicate regions of highest sequence conservation. (B) *In silico* PCR was performed on the regions of interest using the ‘pcr.seq’ function in *mothur* with zero mismatches allowed (*m* = 0). (C) Variance in amplicon sizes produced by *in silico* PCR using the top four primer sets. Each open circle represents a non-redundant sequence for the given region. QCV = Interquartile coefficient of variation. (D and E) Species-level alpha diversity metrics were calculated using the Shannon’s H and Berger-Parker indices for the top four 16S gene regions showing the highest extraction rates following *in silico* PCR. (F) Genus-level breakdown of 16S rRNA gene sequence extraction efficiencies using the top four primer sets tested. Calculations were performed using the ‘extract-reads’ command of the *q2-feature-classifier* in QIIME2, allowing for up to 3 mismatches (*m* = 3) between the forward and reverse primer sequences combined. The lower the extraction efficiency, the higher likelihood that taxa will be underrepresented or potentially missed in a given sample.

Primer pair selection determines which hypervariable region of the 16S rRNA gene is amplified, and thereby can strongly influence the results attained in microbiota studies (53–55). Calculation of pairwise entropy at each nucleotide site showed expected regions of hypervariability among sequences in the *BEEx-FL-refs* data set (Fig. 2A). Two intrinsic limitations to 16S rRNA gene-based microbial identification using current sequencing technologies are primer bias and ambiguity of shorter sequence reads. Accordingly, *in silico* PCR was performed to provide an informative assessment of which routinely used primer sets (Data Set S1D) offer the most valid representation of bacterial community structure based on the sequences present in *BEEx-FL-refs*. Extraction rates varied substantially across hypervariable regions, with primer sets targeting the V1-V3 region performing very poorly (∼40% extraction; Fig. 2B). In contrast, primer sets targeting V3-V4, V4, V4-V5, or V5-V6 regions demonstrated the highest extraction rates during *in silico* PCR (∼90% in each case; Fig. 2B) and were further assessed for their ability to detect and accurately characterize bee-associated taxonomic representatives. Assuming zero mismatches in primer binding, the sequence length of extracted *in silico* amplicons demonstrated minor variance for V4 (95% confidence interval [CI] = 252 to 255 bp; interquartile coefficient of variation [QCV] = 1.19%), V4-V5 (95% CI = 372 to 377 bp; QCV = 1.60%), and V5-V6 (95% CI = 299 to 301 bp; QCV = 1.33%) primer sets (Fig. 2C). The extraction set obtained using the V3-V4 (95% CI = 404 to 431 bp; QCV = 6.52%) primer set also showed minimal variance in the primary amplicon (∼429 bp) but exhibited a multimodal distribution in sequence length with approximately 15% divergence toward shorter secondary amplicons (∼409 bp)—a feature which has the potential to negatively impact some but not all classifier algorithms (33).

Since redundancy of extracted sequences can occur in a hypervariable region-dependent manner (i.e., sequences which are unique at full length can be identical to close relatives when fragmented), we assessed how primer selection may impact common diversity metrics used in bee microbiota studies. On the basis of Shannon’s H index (accounting for species abundance and evenness) and the Berger-Parker index (proportional abundance of the most abundant species, or species dominance), primer sets targeting V4 and V4-V5 regions demonstrated the lowest divergence from species-level alpha diversity of the parent data set (Fig. 2D and E). Conversely, V5-V6-targeting primers seemingly produced the least accurate representation of microbial community structure in terms of alpha diversity. To provide an estimate of how this bias may impart discrepancies between microbiota studies using different primer sets, we performed *in silico* PCR under realistic conditions allowing for incremental mismatches (*m* = 1 to 3) during primer binding (Fig. 2F).

Overall, no “perfect” primer set was identifiable for profiling of bee-associated microbial communities, though for all intents and purposes, V3-V4 primers likely offer the most comprehensive and accurate assessment. V4 primers also demonstrated that they were adequate at capturing bee-associated sequences but cannot distinguish between closely related *Gilliamella* spp. that occur in different bee hosts due to a lack of information density in the shorter spanning sequence length (Fig. 2C) (see Fig. S1A and B in the supplemental material for type strain comparisons). In contrast, V4-V5 primer sets are particularly poor at detecting *Bifidobacterium* and *Bombiscardovia* spp. (complete extraction failure at *m* = 0) which are important microbiota members in corbiculate bees. Whereas V5-V6 primers demonstrated the lowest overall performance, failing to extract many species even after allowing up to *m* = 2 mismatches in primer site binding including that of pathogenic intracellular *Spiroplasma* spp. (Fig. 2F). For inquiries on specific taxa of interest, an extended breakdown table is provided which lists exact values for each of the primer sets tested against all reference sequences in the *BEEx-FL-refs* data set (Data Set S1E).

10.1128/mSystems.00082-21.1 FIG S1 Region-specific effects within the 16S rRNA gene on taxonomic ambiguity and taxonomic classification rates. (A and B) The heatmap plots demonstrate the detectable dissimilarity between each validly published species with respect to 16S rRNA gene sequence identity at each of the variable regions shown. The V3-V4 region is the only sequence region that can distinguish species-level identity comparable to that of the full 16S rRNA gene for both *Gilliamella* and *Lactobacillus* spp. (originally referred to as phylotype members “Gamma-1” and “Firm-5”, respectively). Variable region sequences were extracted *in silico* using the “pcr.seqs” command in mothur, and sequence dissimilarity matrices were calculated using the “DistanceMatrix” function of the DECIPHER package in R. Heatmaps were generated with the gplots package in R and accompanying dendrograms with the “complete” linkage method using the “IdClusters” function of the DECIPHER package in R. (C) Classification was performed in triplicate against the three simulated query sets *simQS-V3V4-i*, *simQS-V3V4-ii*, and *simQS-V3V4-iii* using either untrimmed *BEEx-FL-TS* or trimmed *BEEx-V3V4-TS* training sets. The difference between training sets is most pronounced when bootstrap support is low and, as expected, becomes less apparent as bootstrap support increases and more sequences are also left unclassified. DADA2-NBC was used as the classifier for consistency with previous study benchmarks. Data depict means ± standard deviations and statistics shown for two-way ANOVA with Sidak’s multiple comparisons. Download FIG S1, PDF file, 0.3 MB. Copyright © 2021 Daisley and Reid. 2021 Daisley and Reid. https://creativecommons.org/licenses/by/4.0/ This content is distributed under the terms of the Creative Commons Attribution 4.0 International license.

### Classifier comparisons and selection.

**FIG 3:**
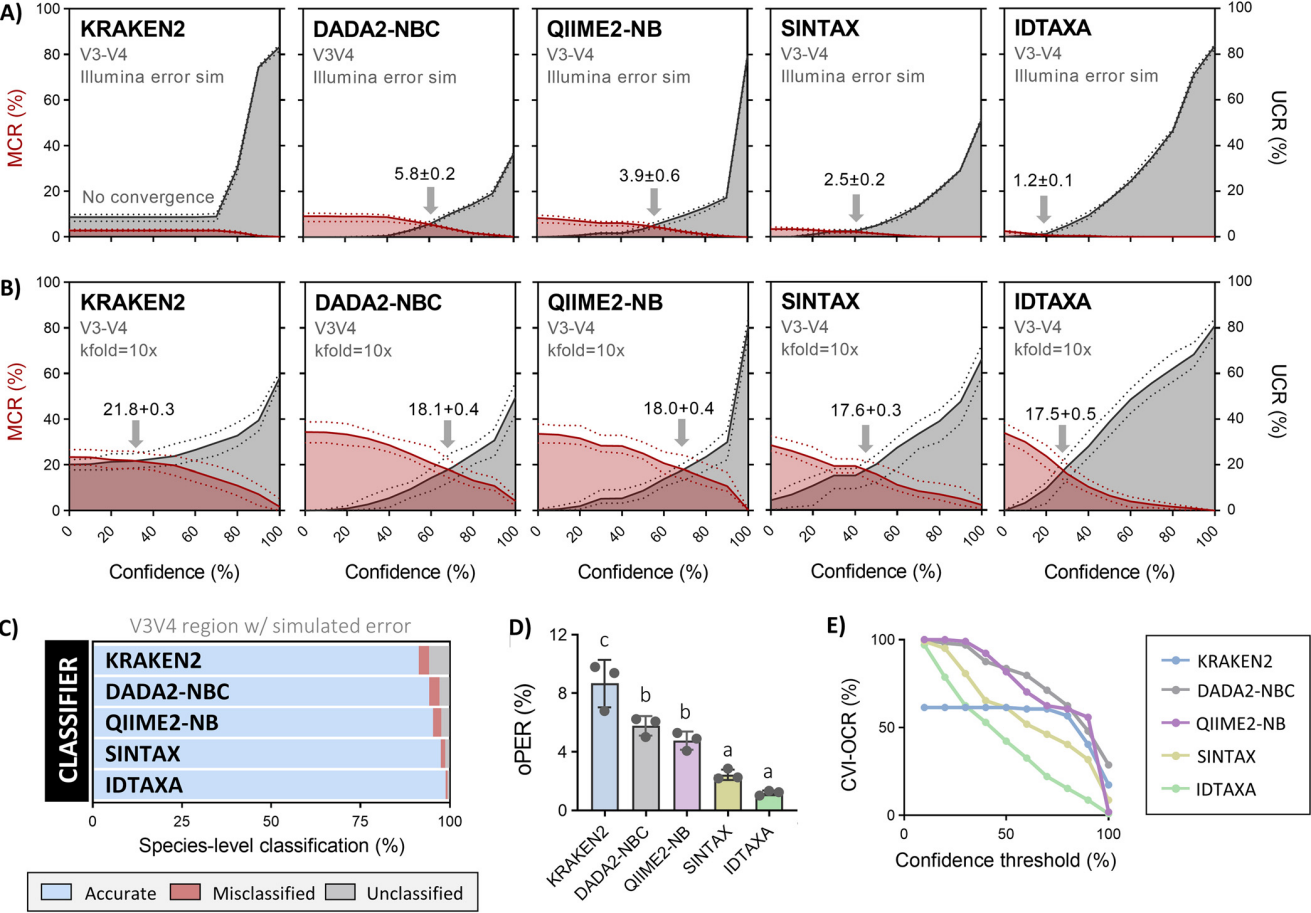
Classifier comparisons against simulated error and novel sequence decisions. (A) *BEEx-V3V4-TS* was used as the training set to classify *n* = 3 randomly sampled test subsets (*simQS-V3V4-i* to *-iii*; 500 sequences each) which were derived from the parent data set but had ∼1% simulated error introduced. (B) *k-*fold cross validation (*k* = 10) tests were performed to assess the ability of each classifier to accurately assign novel sequences in the case when using a training set which does not contain any common sequences with the corresponding test set being classified. Plots represents the species-level misclassification rate (MCR) and underclassification rate (UCR) for the labeled classifier. Default classifier settings were used, and confidence thresholds were set at increasing increments of 10 (across the available range of 0 to 100) to approximate optimal performance error rate (oPER) where MCR ≈ UCR (shown above the gray indicator arrows in each graph). (C) Visual summary of species-level classification rates for the error-simulated query sets. (D) Optimal performance error rate (oPER) comparisons. Data depict means ± standard deviations (error bars) (one-way ANOVA with Tukey’s multiple comparisons) of *n* = 3 classification runs using the *simQS-V3V4-i* to *-iii* data sets. (E) Overclassification rates (OCRs) shown for V3V4-trimmed sequences as determined by cross validation by identity (CVI) using TAXXI benchmark scripts.

Taxonomic classifiers are considered to be of secondary importance compared to the reference database and sequencing technologies used (54), though their impact on study outcome is not negligible and distinct advantages exist, particular in terms of accuracy (50). To evaluate the relevant classifier algorithms, the *BEEx-FL-refs* data set was first trimmed to the hypervariable regions of interest to generate several training sets (*BEEx-FL-TS*, *BEEx-V4-TS*, *BEEx-V3V4-TS*, *BEEx-V4V5-TS*, and *BEEx-V5V6-TS*) which were then converted into a compatible format based on classifier specifications. Recent reports suggest that trimmed training sets offer improvement in performance over their full-length counterparts (1), which we also independently validated in this study can reduce classification error by up to ∼1.5% (Fig. S1C). Next, we compare several classifiers (Fig. 3) including KRAKEN2, SINTAX, IDTAXA, the naïve Bayesian classifier implemented in DADA2 (DADA2-NBC), and the naïve Bayes scikit-learn classifier implemented in QIIME2 (QIIME2-NB) for their ability in accurately annotating query sequences in *simQS-V3V4-i* to *simQS-V3V4-iii*—simulated short-read data sets generated by introducing realistic error rates (∼1%) to bee-associated V3-V4 sequences (randomly sampled from the parent database *BEEx-FL-refs* during *in silico* PCR) using established Mosla Error Simulator (MESA) software (56) (see Materials and Methods section for more details).

Similar to previous reports using human gut and soil sample data sets (50), SINTAX, QIIME2-NB, and DADA2-NBC achieved comparable classification rates and demonstrated a nearly perfect trade-off between decreased true-positive and false-positive annotation rates as confidence thresholds (i.e., bootstrap support cutoffs) increased (Fig. 3A). The mean optimal performance error rate (oPER ± standard error [SE]) for SINTAX, QIIME2-NB, and DADA2-NBC, calculated using the confidence threshold at which sequence misclassification rates (MCRs) and underclassification rates (UCRs) were at their combined lowest, was determined to be 2.4% ± 0.2%, 3.9% ± 0.6%, and 5.8 ± 0.4%, respectively (Fig. 3A). The KRAKEN2 classifier, which has been reported to be faster and more accurate than QIIME2-NB (25), demonstrated very low error rates supporting past accuracy claims but also left many sequences unclassified resulting in the overall worst performance (oPER = 8.7% ± 0.94%; Fig. 3C). Conversely, IDTAXA demonstrated a significantly lower oPER (1.2% ± 0.1%) compared to KRAKEN2, DADA2-NBC, and QIIME2-NB classifiers and trended toward being lower than SINTAX (Fig. 3D). Uniquely, IDTAXA also demonstrated a sharp decline in error rates as bootstrap support increased and had the lowest error rates across all confidence thresholds tested (Fig. 3A to D)—a distinguishing feature potentially explainable by its *de novo* detection of putative mislabeling errors in reference training sets and the ability to automatically correct for spurious query matches (33). To demonstrate robustness, we also performed these same tests on simulated V4, V4-V5, V5-V6, and full-length query sets and show that IDTAXA reliably exhibits the best performance in nearly every case (Fig. S2).

10.1128/mSystems.00082-21.2 FIG S2 Full panel benchmark of classifier performances using simulated sequencing error. Each plot represents the species-level misclassification rate (MCR) and underclassification rate (UCR) for the labeled classifier. For each region evaluated, the *BEEx-FL*, *BEEx-V3V4*, *BEEx-V4*, *BEEx-V4V5*, and *BEEx-V5V6* sequence sets were used as training sets to classify *n* = 3 randomly sampled test subsets (e.g., *BEEx-FLsim01-03*, *BEEx-V3V4sim01-03*, *BEEx-V4sim01-03*, *BEEx-V4V5sim01-03*, and *BEEx-V5V6sim01-03*; 500 unique sequences each set) which were derived from their respective parent data set but had ∼1% simulated error introduced to account for realistic variability due to sequencing error. Only sequences which were classifiable within the region being tested were considered for calculations (i.e., sequences with ambiguous taxonomy due to a lack of sequence dissimilarity in shorter hypervariable region fragments were not counted against classifier scores since this is an inherent limitation of the 16S rRNA gene and not the classifier performance). Default classifier settings were used except for confidence score cutoffs, which were set at increasing increments of 10 (across the available range of 0 to 100) to approximate optimal threshold boundaries where MCR ≈ UCR. Download FIG S2, PDF file, 0.4 MB. Copyright © 2021 Daisley and Reid. 2021 Daisley and Reid. https://creativecommons.org/licenses/by/4.0/ This content is distributed under the terms of the Creative Commons Attribution 4.0 International license.

Under realistic scenarios, the training set will not always possess adequately similar matches to enable species-level classification of all sequences in the query set, which increases the number of decisions made for assigning taxonomy based on lowest common rank (LCR). Thus, we performed *k*-fold cross-validation (57) to stress test the classifiers against novel sequences (i.e., all query sequences were completely absent from the training set). Classifiers unanimously demonstrated substantially higher error rates and worse oPRs during classification of (*k *= 10) V3-V4 query sequence sets, but as in the simulated error test runs, IDTAXA performed best with similar trends existing for the other classifiers (Fig. 3B). However, since *k*-fold and other nonphylogenetically aware cross-validation methods have been criticized as being unrealistic, we also performed cross-validation by identity (CVI) using the TAXXI benchmark which has recently been proposed as a viable solution (50). Consistent with results so far, IDTAXA demonstrated lower CVI overclassification rates (OCRs) at nearly every confidence threshold for the V3-V4 region compared to the other classifiers (Fig. 3E). Once again, all tests were performed on V4, V4-V5, V5-V6, and full-length query sets, with the full panel benchmarks for both *k*-fold cross-validation and CVI provided for completeness (Fig. S3 and Data Set S1F).

10.1128/mSystems.00082-21.3 FIG S3 Full panel benchmark of classifier performances using *k*-fold cross validation. Each plot represents the species-level misclassification rate (MCR) and underclassification rate (UCR) for the labeled classifier when combined with a training set which does not contain any common sequences with the corresponding test set being classified. Only sequences which were classifiable within the region being tested were considered for calculations (i.e., sequences with ambiguous taxonomy due to a lack of sequence dissimilarity in shorter hypervariable region fragments were not counted against classifier scores since this is an inherent limitation of the 16S rRNA gene and not the classifier performance). Test training sets were constructed for the *BEEx-FL*, *BEEx-V3V4*, *BEEx-V4*, *BEEx-V4V5*, and *BEEx-V5V6* sequence sets using the “createFolds” function (*k *= 10) of the *caret* package in R. Each of the 10 training sets was then used to classify the matching test sets with default classifier settings except for confidence score cutoffs, which were set at increasing increments of 10 (across the available range of 0 to 100) to approximate optimal threshold boundaries where MCR ≈ UCR. Download FIG S3, PDF file, 0.4 MB. Copyright © 2021 Daisley and Reid. 2021 Daisley and Reid. https://creativecommons.org/licenses/by/4.0/ This content is distributed under the terms of the Creative Commons Attribution 4.0 International license.

These findings together represent the first comparative report on how different classifier algorithms affect annotation accuracy of bee-associated 16S rRNA gene sequences and independently validate IDTAXA performance claims (33) on the basis of lower error rates and higher total number of accurately classified query sequences.

### Comparisons between BEExact and existing databases.

Based on findings so far, IDTAXA was used as the preferred classifier to determine BEExact performance in comparison with the latest versions of several universal databases (SILVA v138, GTDB r86, and GreenGenes v13.8) as well as the two honey bee (*Apis* spp.)-specific databases, HoloBee v2016.1 (58) and HBDB (23). The latter, HBDB, was modified to included species-level annotations (based on NCBI taxonomy; see Materials and Methods section for full details) for phylotype members that at the time of study were not fully taxonomically characterized. All databases were also trimmed to the 16S rRNA gene region of interest prior to use as training sets (e.g., formatted as SILVA-‘variable region’-TS).

**FIG 4:**
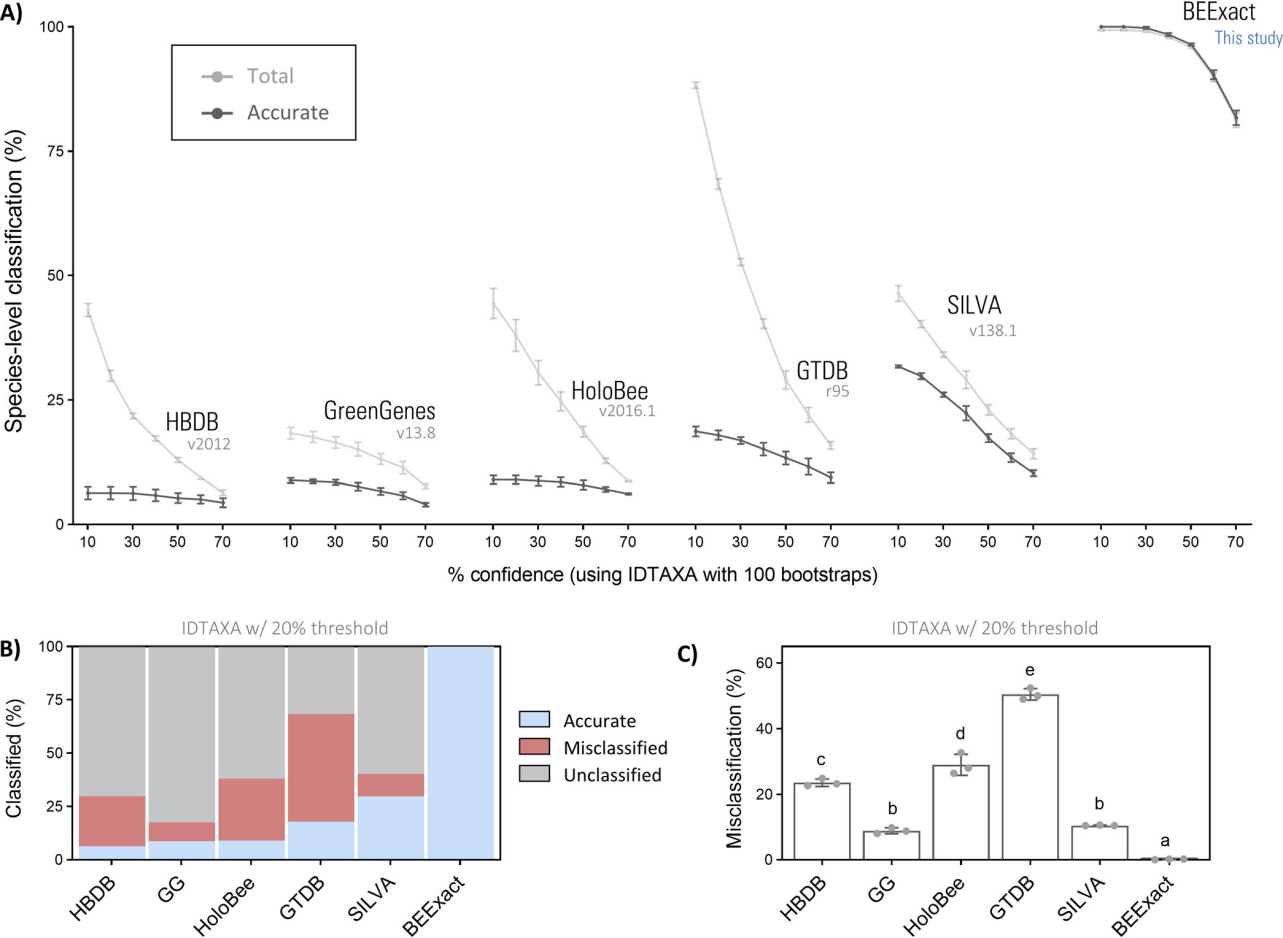
BEExact outperforms against existing databases. (A) Classification rates of V3-V4 simulated reads (i.e., *simQS-V3V4-i* to *-iii* query sets) using IDTAXA with BEExact (*BEEx-V3V4-TS*), Genome Taxonomy Database r95 (*GTDB-V3V4-TS*), Honey Bee Database v20112 (*HBDB-V3V4-TS*), SILVA v138 (*SILVA-V3V4-TS*), GreenGenes v13.8 (*GG-V3V4-TS*), or HoloBee v2016.1 (*HoloBee-V3V4-TS*) training sets. (B) Visual summary of species-level classification rates and (C) error rate comparisons between training sets with IDTAXA (bootstrap cutoff = 20). Error rate data are depicted as means ± standard deviations (one-way ANOVA with Tukey’s multiple comparisons) for *n* = 3 separate classification runs with the *simQS-V3V4-i* to *-iii* query sets.

Using the full-length simulated query sets from previous steps (*simQS-FL-i* to *-iii*), all existing universal database-derived training sets performed well at enabling assignment of taxonomy at higher ranks with total classification rates ranging from ∼95 to 100% for phylum, class, order, and family (Table 2). Consequently, due to the limited reference set sizes of *HBDB-FL-TS* and *HoloBee-FL-TS*, total classification rates were ∼20 to 30% lower at the family rank and higher (Table 2). At the genus and species level for existing databases, *HoloBee-FL-TS* and *HBDB-FL-TS* classified most honey bee-specific taxa present in the query set (as their original purpose intended), whereas S*ILVA-FL-TS* and *GTDB-FL-TS* displayed the highest overall classification rates at both ranks. Notably, none of the training sets tested besides *BEEx-FL-TS* could accurately achieve beyond ∼30% species-level classification at any confidence threshold (Fig. 4A and B).

**TABLE 2:** Demonstrative full lineage results using V3V4 training sets on *simQS-V3V4-i* to *-iii* query sets

Database	% total classified, % misclassified, or % accurately classified (mean ± SE)^*a*^					
	Phylum	Class	Order	Family	Genus	Species
% total classified						
BEExact	100.0 ± 0.0	100.0 ± 0.0	100.0 ± 0.0	100.0 ± 0.0	100.0 ± 0.0	100 ± 0.0
SILVA v138	100.0 ± 0.0	100.0 ± 0.0	100.0 ± 0.0	99.4 ± 0.1	95.3 ± 0.1	40.3 ± 0.4
GTDB r95	99.3 ± 0.1	99.3 ± 0.07	98.7 ± 0.2	97.5 ± 0.2	89.0 ± 0.4	68.4 ± 0.6
HoloBee v2016	89.2 ± 0.4	88.8 ± 0.4	83.5 ± 0.4	80.7 ± 0.7	75.4 ± 0.9	38.0 ± 1.8
GG v13.8	100.0 ± 0.0	100.0 ± 0.0	99.8 ± 0.1	95.0 ± 0.7	79.0 ± 0.6	17.5 ± 0.6
HBDB v2012	87.4 ± 0.2	85.3 ± 0.2	70.3 ± 0.3	61.3 ± 0.8	53.73 ± 0.8	29.9 ± 0.6

% misclassified						
BEExact	0.0 ± 0.0	0.0 ± 0.0	0.0 ± 0.0	0.0 ± 0.0	0.0 ± 0.0	0.1 ± 0.1
SILVA v138	5.6 ± 0.5	1.1 ± 0.1	3.5 ± 0.2	4.3 ± 0.2	10.2 ± 0.2	10.5 ± 0.1
GTDB r95	5.9 ± 0.7	24.4 ± 0.2	18 ± 0.7	19.7 ± 0.8	10.7 ± 0.4	50.5 ± 1.0
HoloBee v2016	24.9 ± 0.4	10.5 ± 0.4	18.4 ± 0.4	13.9 ± 0.9	22.9 ± 1.0	29.0 ± 1.9
GG v13.8	26.1 ± 0.4	12.9 ± 0.4	48.4 ± 0.7	13.1 ± 0.4	12.07 ± 0.4	8.9 ± 0.5
HBDB v2012	23.3 ± 0.4	7.5 ± 0.4	11.4 ± 0.4	13.1 ± 0.2	20.1 ± 0.8	23.5 ± 0.7

% accurately classified						
BEExact	100.0 ± 0.0	100.0 ± 0.0	100.0 ± 0.0	100.0 ± 0.0	100.0 ± 0.0	99.9 ± 0.1
SILVA v138	94.4 ± 0.5	98.9 ± 0.1	96.5 ± 0.2	95.1 ± 0.3	85.1 ± 0.2	29.8 ± 0.3
GTDB r95	64.1 ± 0.2	77.9 ± 0.5	58.9 ± 0.1	48.2 ± 0.6	33.6 ± 0.4	6.3 ± 0.7
HoloBee v2016	64.3 ± 0.2	78.3 ± 0.8	65.1 ± 0.7	66.8 ± 0.2	52.5 ± 0.3	9.0 ± 0.5
GG v13.8	93.5 ± 0.7	74.9 ± 0.2	80.7 ± 0.6	77.8 ± 0.9	78.3 ± 0.8	17.9 ± 0.5
HBDB v2012	73.9 ± 0.4	87.1 ± 0.4	51.4 ± 0.8	81.9 ± 0.9	66.9 ± 0.6	8.7 ± 0.2

a All values were obtained using IDTAXA (bootstrap cutoff = 20).

In contrast to the observed trends for total classification rates, the accompanying error rates for *GG-FL-TS*, *SILVA-FL-TS*, and *GTDB-FL-TS* were considerably variable across higher taxonomic ranks (family and above), which is counterintuitive to the relatively lower error rates displayed at the genus level for these training sets (Table 2). Specifically, further inspection revealed that the MCR associated with *GTDB-FL-TS* was strikingly higher than all other training sets tested, with the effect rapidly lessening as IDTAXA confidence thresholds were incrementally raised (Fig. 4).

These findings, alongside the fact that IDTAXA automatically corrects for most taxonomic branching order disagreements, suggests that these higher rank errors are likely due to either database-specific artifacts from custom branching order of taxonomic lineages (16) or the systematic propagation of outdated lineage names from the latest Bergey’s taxonomy manual (59), which was last updated in 2012. Visual inspection of the data confirmed this, in part, demonstrating that despite identical genus- or species-level classifications of a given sequence between databases, there were several discrepancies in identity at the phylum (e.g., *Actinobacteria* → *Actinobacteriota* and *Bacteroidetes* → *Bacteroidota*), class (e.g., *Betaproteobacteria* → *Gammaproteobacteria*), order (e.g., *Pseudonocardiales* → *Corynebacteriales*, *Rhodospirillales* → *Acetobacterales*, *Orbales* → *Enterobacterales*, *Neisseriales* → *Betaproteobacteriales*, and *Bifidobacteriales* → *Actinomycetales*), and family (e.g., *Orbaceae* → *Enterobacteriaceae*, *Flavobacteriaceae* → *Weeksellaceae*, *Leuconostocaceae* → *Lactobacillaceae*, *Yersiniaceae* → *Enterobacteriaceae*, *Morganellaceae* → *Enterobacteriaceae*, and *Paenibacillaceae* → *Brevibacillaceae*) levels for many database references. In the case of GTDB, many of these discrepancies are likely the result of recent standardized taxonomic revisions based on whole-genome phylogenomics (60). Many advantages exist for restructuring taxonomic lineage by way of marker gene conversion based on whole-genome data, but unidentified contamination remains a concern (61). Indeed, official GTDB documentation (https://data.ace.uq.edu.au/public/gtdb/data/releases; see “FILE_DESCRIPTIONS”) states that contaminating sequence fragments in the database can cause incongruent taxonomic assignment in certain cases. Together, this may readily explain the relatively high rate of species-level classification using *GTDB-FL-TS* (Table 2 and Fig. 4A and B) alongside the pronounced error rate of ∼50% (confidence threshold = 20% with IDTAXA), especially compared to the significantly lower error rates observed using other universal database-derived *GG-FL-TS* (∼9%) or *SILVA-FL-TS* (∼11%) training sets (Table 2 and Fig. 4C). Despite the latter similarities, *SILVA-FL-TS* offered the most balanced profile among the existing classifiers and accurately classified over threefold-more sequence than *GG-FL-TS* (Table 2). In accordance with these findings, SILVA-derived training sets were used for all subsequent comparative validation experiments as a measurable reference point on which to gauge BEExact performance against the best leading database in existence.

### Validating BEExact performance on published data sets.

As a demonstration of its ultimate purpose, we assessed the performance of BEExact on classifying high-throughput 16S rRNA gene sequencing data derived from 32 independent literature sources in which bee host-associated microbial communities were sampled (Table 3).

**TABLE 3:** List of 16S rRNA gene sequencing data sets used for validation in this study

Accession no. (reference)	Pipeline (reference)	Method	Region	Classifier algorithm	Reference database	No. of reads
PRJNA554741 (96)	UPARSE (v7.1)	OTU_97_	V3-V4	RDP-NBC	SILVA v123	1,378,161^*c*^
PRJNA304949 (97)	QIIME (v1.7.0)	OTU_97_	V3-V4	UPARASE	SILVA v119	1,509,164^*b*^
PRJNA348791 (65)	QIIME 1.7.0	OTU_97_	V3-V4	BLASTN	SILVA v119	1,882,956^*b*^
PRJNA382070 (98)	MALT v0.3	OTU_97_	V3-V4^*e*^	MEGAN	N/A	21,676,026^*c*^
PRJNA517228 (99)	QIIME v1.7	OTU_97_	V3-V4	RDP-NBC	SILVA v128	2,341,256^*b*^
CRA001462 (100)	QIIME^*a*^	OTU_97_	V3-V4	UCLUST	SILVA^*a*^	2,249,189^*b*^
PRJEB22577 (101)	QIIME v1.8	OTU_97_	V3-V4	SINTAX	RDPv16 + SILVAv128 + custom db^*d*^	33,879,177^*b*^
PRJEB25500 (64)	USEARCH v9.0	OTU_97_	V3-V4	SINTAX	RDPv16 + SILVAv128 + custom db^*d*^	31,212,532^*b*^
PRJEB27239 (102)	USEARCH v8.1	OTU_97_	V4	UCLUST	RDPv16	5,126,605^*c*^
PRJEB27223 (103)	QIIME^*a*^	OTU_97_	V4	RDP v2.11	GG v13.5	5,258,089^*c*^
PRJNA610196 (104)	DADA2 v1.8	ASV	V4	DADA2-NBC	SILVA v132 + HBDB	1,685,442^*c*^
PRJNA371284 (105)	QIIME v1.9.1	OTU_97_	V4	BLASTN	GenBank nt/nr	3,932,593^*c*^
PRJNA491200 (106)	mothur v1.39.5	OTU_97_	V4	RDP-NBC	RDP v15 + SILVA v128 + GenBank nt/nr	862,843 ^*c*^
PRJNA432210 (107)	QIIME v1.9.1	OTU_97_	V4	N/A	Custom db^*f*^	2,643,811^*c*^
PRJNA589199 (108)	VSEARCH^*a*^	OTU_97_	V4	SINA	SILVA v132	322,079^*c*^
PRJEB23223 (109)	LotuS^*a*^ (110)	OTU_97_	V4	RDP-NCB	GreenGenes^*a*^ + SILVA^*a*^	14,474,484^*c*^
PRJEB23224 (111)	LotuS^*a*^	OTU_97_	V4	RDP-NCB	SILVA^*a*^ + GG^*a*^ + beetax	23,340,731^*c*^
PRJNA429464 (112)	QIIME v1.9.0	OTU_97_	V4	SINA	SILVA^*a*^ + custom placement^*e*^	399,656^*c*^
PRJNA225925 (113)	QIIME v1.7	OTU_97_	V4	BLASTN	GenBank nt/nr	497,260^*c*^
PRJNA483763 (43)	QIIME 1.9.1	OTU_97_	V4	UCLUST	SILVA v128	2,138,393^*c*^
PRJNA432211 (114)	QIIME 1.9.1	OTU_97_	V4	UCLUST + BLASTN	GG v13.8 + GenBank nt/nr	2,444,254^*c*^
PRJNA578869 (115)	DADA2 v1.12.1	ASV	V4	DADA-NBC + BLASTN	SILVA^*c*^ + GenBank nt/nr	3,011,306^*c*^
dryad.33518g8 (116)	QIIME2	ASV	V4	QIIME2-NB + BLASTN	GenBank nt/nr	16,866,658^*b*^
PRJNA309422 (10)	QIIME v1.9.1	OTU_97_ OTU_99.5_	V4	UCLUST + BLASTN	SILVA v119 + GenBank nt/nr	14,500,577^*c*^
PRJNA596093 (117)	mothur v1.40.5	V4	RDP-NBC	BGM-Db	3,378,791^*c*^
PRJNA530255 (118)	QIIME v1.9.1	OTU_97_	V4-V5	UCLUST + BLASTN	GG v13.8 + SILVA v132	2,446,119^*b*^
PRJNA529891 (119)	USEARCH^*a*^	OTU_97_	V4-V5	UCLUST	SILVA v132	3,378,791^*c*^
PRJEB27718 (74)	VSEARCH^*a*^	OTU_97_	V4-V5	UCLUST + PANAM	SILVA^*a*^	9,770,720^*b*^
PRJNA485519 (120)	QIIME2 + DADA2	ASV	V5-V6	QIIME2-NB	SILVA^*a*^ + GenBank nt/nr	2,258,202^*b*^
PRJNA436176 (121)	QIIME2 + DADA2	ASV	V5-V6	QIIME2-NB	SILVA^*a*^ + GenBank nt/nr	2,632,180^*b*^
PRJNA464035 (122)	QIIME2 + DADA2	ASV	V5-V6	QIIME2-NB	SILVA v128	6,393,486^*b*^
PRJNA454884 (123)	QIIME2 + DADA2	ASV	V5-V6	QIIME2-NB	GG v13.8	10,676,029^*b*^
Total no. of reads	234,567,560					

a Version or release number not specified for software used.

b Illumina MiSeq paired-end reads (2 × 250 bp).

c Illumina MiSeq paired-end reads (2 × 300 bp).

d Custom reference database consisted of 910 sequences retrieved from NCBI which were assigned taxonomy based on reconstructed phylogeny using published reference sequences (identity thresholds not provided).

e Custom placement of sequences using RAxMLv7.4.2 software.

f Custom local database, details undisclosed.

Following retrieval from the SRA database, all data sets were processed similarly through the DADA2 pipeline resulting in a nonredundant set of 6,847 total V3-V4 region ASVs (*nrQS-V3V4*), 12,614 total V4 region ASVs (*nrQS-V4*), 729 total V4-V5 region ASVs (*nrQS-V4V5*), and 3,554 total V5-V6 region ASVs (*nrQS-V5V6*) before removal of contaminants including ASVs originating from mitochondria, chloroplast, and host bee genomes which were not considered in classification rate calculations (Data Set S1G and H). To provide guidance in future studies, we first evaluated a single-region subset of ASVs from the largest data set (*nrQS-V4*) to determine how sequence depth impacts the overall quality and comprehensiveness of surveying bee-associated microbial communities. We determined that the total number of detectable ASVs per study was strongly and positively correlated (*R*^2^= 0.9337) with per sample read counts (i.e., read depth; Fig. S4A and B).

10.1128/mSystems.00082-21.4 FIG S4 A minimum sequencing depth is required to obtain a representative assessment of microbial community structure. (A) UpSet plot visualizing the intersections of unique ASVs in an eight-data set subsample from *nrQS-V4* (PRJEB23223 [JJ], PRJNA309422 [WK], PRJNA429464 [JP], PRJNA432210 [EM], PRJNA483763 [KR], PRJNA491200 [BS], PRJNA578869 [LK], and PRJNA610196 [BD]). Plot generated the UpSet package in R. (B) Unique ASVs per study as a function of mean read depth per sample for each of the published data sets. (C) Unique ASVs per simulated run (generated using the ART command line “art_illumina”) as a function of read depth. Semi-log line of fit of best fit (solid black line) is shown with 95% confidence interval bands (dotted gray lines) calculated using the least squares regression method. *R*^2^ is the adjusted coefficient of determination for nonlinear regression. Download FIG S4, PDF file, 0.2 MB. Copyright © 2021 Daisley and Reid. 2021 Daisley and Reid. https://creativecommons.org/licenses/by/4.0/ This content is distributed under the terms of the Creative Commons Attribution 4.0 International license.

**FIG 5:**
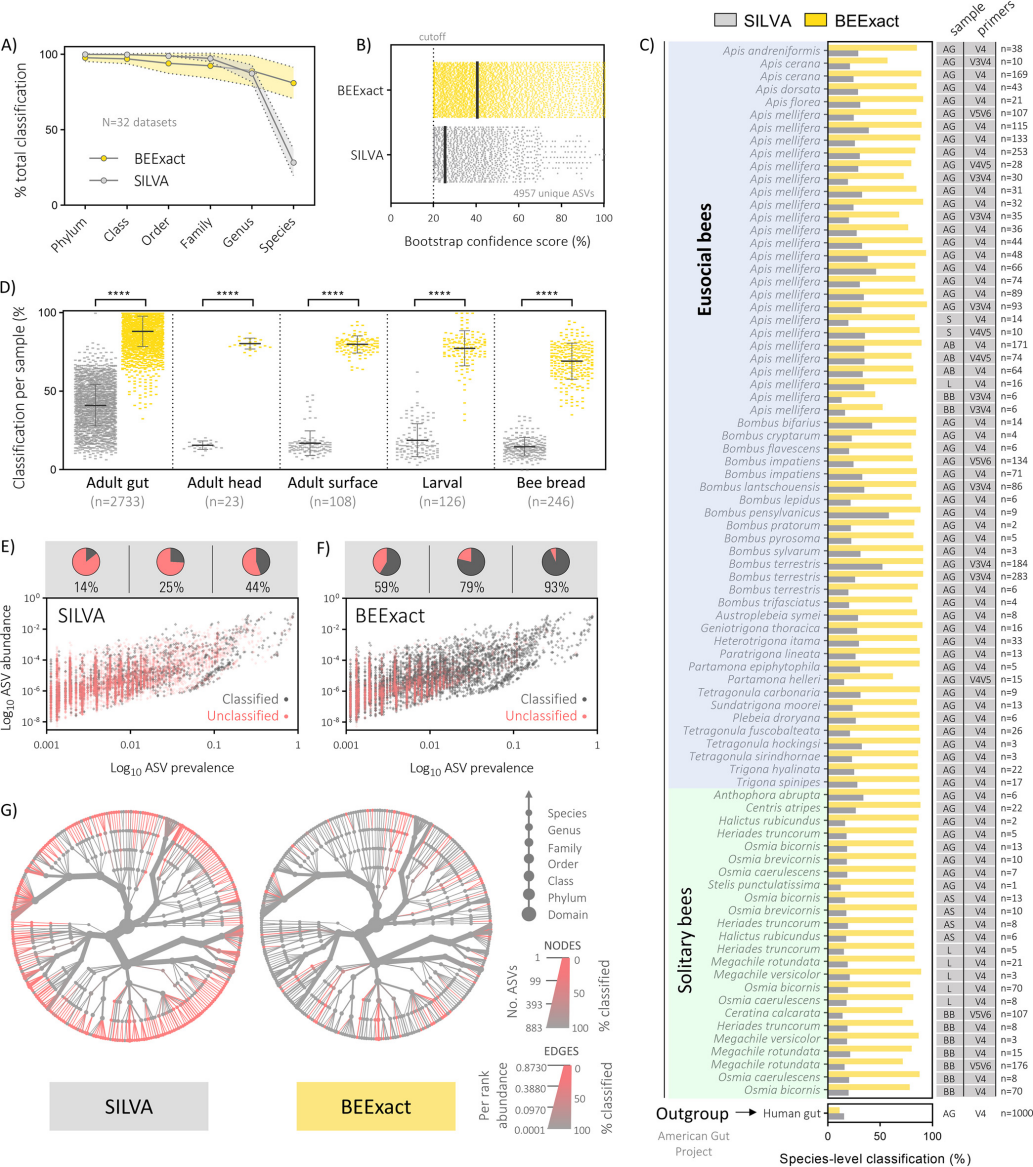
BEExact classifies more ASVs and at higher confidence compared to the widely implemented SILVA database. (A) Overall classification rates at each taxonomic level for all data sets evaluated. Data depict means ± standard deviations at each level for *n* = 32 data sets with statistics shown for two-way ANOVA with Tukey’s multiple comparisons. (B) IDTAXA bootstrap confidence scores on the total set of 4,957 unique ASVs from all data sets combined. The dotted line showing the cutoff (20%) used for all other comparisons shown. (C and D) Classification rates broken down by bee species (grouped by eusocial or solitary type membership) (C) and by sample type irrespective of background bee type (D). Data depict means ± standard deviations per sample classified in each of the categories shown (two-way ANOVA with Tukey’s multiple comparisons). (E and F) Scatterplots demonstrate that BEExact outcompetes SILVA more often in assigning taxonomy to ASVs found at either high prevalence or abundance across all data sets evaluated. Nested visualization plots above show how classification rates change based on differences in ASV prevalence. (G) Heat trees display the weighted classification rates across the entire taxonomic lineage for the top ASVs after collapsing to species-level identity. A cutoff of 1% prevalence or 0.01% abundance was applied to show only the most relevant bee-related taxa while minimizing transient environmental taxa. Abundance was adjusted by normalizing for 16S rRNA copy number differences between taxon groups. AG, adult gut; AH, adult head; S, surface; L, larvae; BB, bee bread.

To confirm that this was a product of sequencing instrument limitations, rather than real variation in microbial diversity of the bee samples between different studies, we utilized an established next-generation sequence simulator (ART [62]) to emulate several MiSeq runs at various read depths using a subset of *in silico*-extracted V4 sequences from *BEEx-FL-refs* (total of 718 unique sequences used as inputs). Based on mimicked error rates calculated from sequencing data evaluated in this study (Fig. S5), less than half of the 718 unique input sequences were detectable by the denoising algorithm implemented in DADA2 at a per sequence read depth of 32,000. As read depth doubled to 64,000, approximately 70% of ASVs were detectable, and at a read depth of 256,000, >80% of ASVs were detectable (Fig. S4C). These trends in the simulated data sets strongly recapitulated empirical observations (Data Set S1G to J; Fig. 5B) and suggest that a majority (approximately 50% or more) of rare or low-abundance bee host-associated sequence variants are likely missed in studies sampling at a read depth of <50,000 reads per sample. Corroborating the reported importance of sequencing depth on characterization of microbial communities (63), the number of ASVs shared between any two or more data sets (i.e., overlapping redundancy of ASVs) was directly related to the total number of ASVs detected in any one data set being compared (Fig. S4A).

10.1128/mSystems.00082-21.5 FIG S5 Quality profiles for real and simulated 16S rRNA gene sequencing runs. (A to I) Visualization of quality profiles for forward and reverse reads were generated in R using the “plotQualityProfile” function of DADA2. The black underlying heatmap depicts the frequency of each score at each base position. The quality profiles for the simulated run (I) are based on learned error rates from the real data sets (A to H) and were calculated using the ART command line tool “art_profiler_illumina.” Data are representative of an eight-data set subsample from *nrQS-V4* (containing PRJEB23223 [JJ], PRJNA309422 [WK], PRJNA429464 [JP], PRJNA432210 [EM], PRJNA483763 [KR], PRJNA491200 [BS], PRJNA578869 [LK], and PRJNA610196 [BD] sequence reads). Download FIG S5, PDF file, 0.7 MB. Copyright © 2021 Daisley and Reid. 2021 Daisley and Reid. https://creativecommons.org/licenses/by/4.0/ This content is distributed under the terms of the Creative Commons Attribution 4.0 International license.

Next, using the IDTAXA classifier, we assessed how reference sequence differences (i.e., BEExact versus SILVA training sets trimmed at each relevant 16S rRNA gene region) impacted overall taxonomic assignment of ASVs found in *nrQS-V3V4*, *nrQS-V4*, *nrQS-V4V5*, and *nrQS-V5V6*. Similar performances were exhibited on a per study basis by all BEExact and SILVA training sets when considering mean ± SE classification rates at the phylum (97.8% ± 0.5% versus 99.01% ± 0.1%; *P* = 0.9666), class (97.02% ± 0.6% versus 99.8% ± 0.1%; *P* = 0.8105), order (94.13% ± 1.2% versus 99.0% ± 0.2%; *P* = 0.0728), family (92.5% ± 1.5% versus 97.4% ± 0.4%; *P* = 0.0740), and genus (89.2% ± 1.8% versus 87.5% ± 1.0%; *P* = 0.9949) levels (Fig. 5A). However, at the species level, BEExact enabled strikingly higher classification rates compared to SILVA (81.0% ± 1.8% versus 28.4% ± 1.6%; *P* < 0.0001).

Since the true taxonomy of ASVs is unknown, classifier confidence thresholds were used as a proxy to gauge the certainty at which taxonomic predictions were made. BEExact produced significantly higher overall mean ± SE confidence scores for species-level classifications compared to SILVA (40.59% ± 0.32% versus 25.5% ± 0.18%; *P* < 0.0001; Fig. 5B, Data Set S1I and J).

Breakdown of classification rates after accounting for background differences in bee host and sample type also demonstrated that BEExact outperformed SILVA in all instances (Fig. 5C and D). Notably, SILVA demonstrated a general trend toward higher classification on samples from eusocial corbiculate bee hosts rather than those from solitary bee origin—an effect potentially due to a higher sequence representation associated with the former as a result of the extensive characterization of social bee gut microbial communities (52). Nonetheless, two of the lowest classification rates based on bee host using either training set came from A. mellifera bee bread and Apis cerana adult gut samples (derived from BioProject accession no. PRJEB25500 [64] and PRJNA348791 [65]), respectively). Thus, this means either that there are still certain novel corbiculate bee-associated taxa awaiting to be discovered or that these studies experienced heavy contamination from environmental sequences.

Abundance and prevalence cutoffs are frequently implemented during data analysis of microbiota studies to eliminate noise and improve data set comprehension, though thresholds are generally chosen arbitrarily. As a reference point for future microbiota studies on bee hosts, we evaluated a series of relative abundance and prevalence cutoffs (calculated for each data set separately) to determine whether there may be an approximate optimal range determinable based on classification likelihood. For BEExact, classification rates sequentially improved with ascending abundance thresholds of 0.001% to 0.1%, which support its niche habitat (bee host) specificity (Fig. S6). Whereas for SILVA, improvement in classification rates occurred after only the highest cutoff (0.1%), which can be expected simply based on the sheer reduction in classifiable sequences (Fig. S6B). Prevalence thresholds demonstrated a similar trend (Fig. S6C and D), and importantly appear to be better suited for data set noise reduction based on visualization of these relationships shown in the prevalence-abundance scatterplots in Fig. 5E and F.

10.1128/mSystems.00082-21.6 FIG S6 Effects of abundance and prevalence cutoffs as they relate to overall taxonomic classification of ASVs. (A) SILVA-derived and (B) BEExact-derived training sets used with IDTAXA (confidence threshold = 20%) to classifying ASVs from *nrQS-V3V4*, *nrQS-V4*, *nrQS-V4V5*, and *nrQS-V5V6* query sets at various abundance cutoffs applied at the level of the data set. (C) SILVA-derived and (D) BEExact-derived training sets used with IDTAXA (confidence threshold = 20%) to classifying ASVs from *nrQS-V3V4*, *nrQS-V4*, *nrQS-V4V5*, and *nrQS-V5V6* query sets at various prevalence cutoffs applied at the level of the data set. Data depict mean ± standard deviation species-level classification for *n* = 32 data sets. Statistics are shown for Kruskal-Wallis tests with multiple comparisons using Benjamini and Hochberg false discovery rate (FDR). *, *P* < 0.05; **, *P* < 0.01; ****, *P* < 0.0001; ns, not significant. Download FIG S6, PDF file, 0.1 MB. Copyright © 2021 Daisley and Reid. 2021 Daisley and Reid. https://creativecommons.org/licenses/by/4.0/ This content is distributed under the terms of the Creative Commons Attribution 4.0 International license.

Specifically, when considering only ASVs found at a prevalence of >1.0% in any given data set, there is never an instance when applying additional abundance cutoffs would yield better classification rates without concurrently eliminating a large majority of ASVs found with a relative abundance between 0.0001 and 0.01% (Fig. 5F). In contrast, applying an abundance cutoff of 0.00001% favorably avoids the large undercut of ASVs (mostly classified by BEExact) found at low abundance and high prevalence, while reducing low-abundance ASVs which BEExact was unable to classify, and thus likely represent environmental contaminants or transient taxa. From these observations and assuming an adequate sample size, a combined prevalence cutoff of ≤0.05% (frequency ≤ 5 × 10^−4^) and abundance cutoff of ≤0.00001% (frequency ≤ 10^−7^) appear justified for general purposes. Taxonomic heat trees for BEExact and SILVA in Fig. 5G display the phylogenetic relatedness of ASVs remaining unclassified after applying the aforementioned cutoffs. Visual inspection demonstrated that despite classifying far more ASVs at the species level, BEExact left twice as many taxon groups (12 versus 6) completely unclassified at the family level or higher (i.e., no lower common rank members in any of the lineage were classified) compared to SILVA (Fig. 5G).

### Probing unclassified ASVs to determine applicability as additional database references.

**FIG 6:**
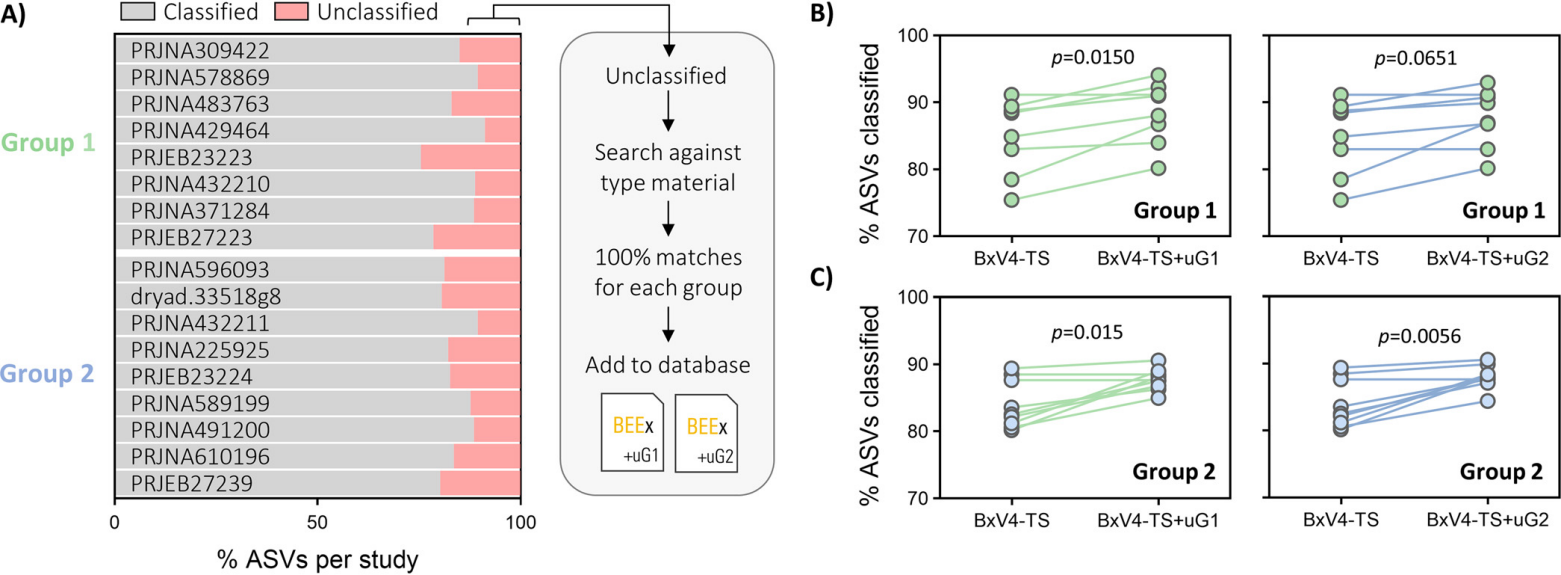
Probing unclassified ASVs to determine applicability as additional database references. Shown is a subset of the V4-16S rRNA gene sequencing data sets evaluated in this study, which were randomly divided into two groups for demonstrative purposes. (A) The bar plot on the left depicts species-level classification rates for each data set using *BEEx-V4-TS* with IDTAXA classifier (bootstrap cutoff = 20%). The flowchart on the right shows the steps taken in supplementing *BEEx-V4-TS* with unmatched ASVs (i.e., unclassified ASVs from either group 1 or 2) to create *BEEx-V4-TS+uG1* and *BEEx-V4-TS+uG2*, respectively. (B and C) Before-after plots show that added sequences in *BEEx-V4-TS+uG1* can increase the classification rates on parent data sets from which they were derived (group 1) as well as independent data sets from which they were not derived (group 2). Similar trends are displayed for *BEEx-V4-TS+uG2*. Individual data points represent total classification rates per study (*n* = 8 for group 1; *n* = 9 for group 2) with statistics shown for two-way ANOVA with Tukey’s multiple comparisons. IDTAXA (bootstrap cutoff = 20%) was used for all comparisons. Green symbols indicate that group 1 data sets are being classified, while blue symbols indicate that group 2 data sets are being classified. The line color indicates which group the unmatched sequences in the training set being compared came from (*BEEx-V4-TS+uG1 *= green, *BEEx-V4-TS+uG2 *= blue).

If the identified groups of unclassified ASVs were indeed derived from bee host-associated microbial communities, then it could be expected that inclusion of sequence representatives in BEExact would further improve classification rates on additional, independent, 16S rRNA gene sequencing data sets derived from bees. To test this theory and demonstrate proof of principle, we randomly broke up the *nrQS-V4* data set (largest single-region sample size and most ASVs of those evaluated) into two groups irrespective of background bee host, sample type, or any other discriminating data set feature (Fig. 6A). ASVs from the first group which were left unclassified by BEExact but matched unambiguously at 100% identity to type material were then added (with annotated taxonomy) to the region-specific BEExact training set to create *BEEx-V4-TS+uG1*.

As expected, when reclassifying ASVs from the first group, using the training set containing the additional annotated reference sequences derived from the same group (*BEEx-V4-TS+uG1*) significantly improved mean ± standard deviation (SD) classification rates (88.5% ± 4.7% versus 84.9% ± 5.6%, respectively) compared to the original training set *BEEx-V4-TS* (Fig. 6B). Next, the same training sets were used to classify the independent set of ASVs from the second group, which showed that *BEEx-V4-TS-uG1* once again exhibited significantly higher classification rates (87.7% ± 1.6% versus 84.0% ± 3.6%, respectively) compared to the original training set *BEEx-V4-TS* (Fig. 6C). For completeness, we also evaluated analogous comparisons using the complementary set of unclassified ASVs in the second group, which when added to the original *BEEx-V4-TS* demonstrated nearly identical patterns of ASV classification improvement for both groups (Fig. 6B and C). On the basis of these findings, we performed a thorough search for all data sets that were evaluated in this study and then supplemented sequence representatives of ASVs with matching criteria (i.e., ≥0.00001% abundance and ≥1.0% prevalence in any data set with unambiguous 100% match to type material) to the final database for maximized performance.

BEExact is publicly available at https://github.com/bdaisley/BEExact and preformatted for seamless integration with IDTAXA (33) as well as the classifiers implemented in DADA2 (27) and QIIME2 (28) pipelines, but can be adapted for use with any classifier permitting customized databases.

## DISCUSSION

This study demonstrated the utilization of a novel method for generating a host-tailored metataxonomic reference database, which when applied to bees, permitted significantly improved species-level classification of 16S rRNA gene sequencing ASVs derived from bee-associated microbial communities. The most notable advancement in this regard is massively improved data set resolution and ability to draw meaningful conclusions based on accurate profiling of taxonomic structure and composition. Furthermore, several sets of primers and classifier algorithms were compared as were the effects of ASV prevalence and abundance cutoffs, which together may provide a useful point of reference for future studies investigating host-microbe interactions in a broad range of bee species.

Evaluation of primer sets commonly used for 16S rRNA gene sequencing demonstrated that V3-V4, V4, V4-V5, and V5-V6 targeting primers offered the highest raw capture rates for bee-associated sequences found in *BEEx-FL-refs* (∼90% for each; Fig. 2B). However, discrimination against taxonomic groups (i.e., identity of noncaptured sequences) varied substantially. When allowing no mismatches in primer binding (*m* = 0), V3-V4 and V4 *in silico* PCR amplicon sets showed the least divergence from originating sequence population based on species-level alpha diversity metrics (Fig. 2D and E). In comparison to other primer sets tested, this suggests V3-V4 and V4 primer sets offer a balanced primer bias and a more representative assessment of true taxonomic composition for bee-associated taxa. However, while V4 primers may be adequate for certain bee hosts with simple microbiota profiles, the information-rich ASVs produced by V3-V4 primers offer considerably higher resolving power in distinguishing between closely related taxa (see Fig. S1 in the supplemental material). Thus, V3-V4 primers which are the gold standard for characterizing plant microbial communities (66) should also be considered the first choice in bee microbiota investigations to facilitate field-wide standardization, thereby enabling both cross-study and cross-host comparisons.

Consistently, V4-V5 and V5-V6 failed to capture any sequence representatives from the genera *Bifidobacterium* and *Bombiscardovia* (V4-V5) or *Apibacter* (V5-V6), all three of which are important core microbiota members in corbiculate bees (52). Notably, primer sets unanimously performed poorly in capturing sequences from *Spiroplasma*, *Micrococcus*, and *Cutibacterium* genera (Fig. 2A to F). Based on simulated modeling of primer binding site promiscuity (*m* = 1 to 3 mismatches; Fig. 2F) as well as empirical findings from the published data sets (Data Set S1G to J) though, most of these taxa were detectable by the primer sets to various degrees. Nonetheless, given that Spiroplasma apis and Spiroplasma melliferum are well-known intracellular parasites of bee hosts (67) and the latter two genera are opportunistic entomopathogens (68, 69), future disease-related investigations may consider additional methods for evaluating these bacteria. Likewise, retrospective analysis may be considered for other pathogens of interest on a primer set-dependent basis to potentially detect hidden or sublethal infections that went unnoticed or were underrepresented due to sequence capture biases (see Data Set S1E in the supplemental material for exact bias predictions of each primer set).

The findings also bring to light the intriguing fact that certain primer sets can detect microsporidia relevant to bee health (Data Set S1G and H and Fig. S7). This includes Nosema ceranae, Nosema apis, and Nosema bombi which are considered amitochondriate (70) and possess 5S, 16S, and 18S rRNA genes (71) unlike that of most other fungal species that have 5.8S, 18S, and 25S rRNA genes. Despite the fact that phylogenetic studies and epidemiological investigations have used the 16S rRNA gene of microsporidia in diagnostic tests over a broad host range (72, 73), it appears largely underutilized in bee microbiota studies and even in those studies focusing on nosemosis (74, 75). In one of these associated data sets evaluated (BioProject accession no. PRJEB27718 [74]; data not made available for the other [75]), we detected three ASVs consistently present across samples that were unambiguously classifiable as N. ceranae (#00025-00027 in *nrQS-V4V5*; Data Set S1G and H). Nearly all data sets constituting *nrQS-V4* also contained ASVs from *Nosema* spp. (Fig. S7). A reasonable assumption is that these sequences may have gone unnoticed until now due to similarities with plant mitochondrial contaminants, though maximum likelihood phylogenetic analysis strongly supports distinct branching order (Fig. S8) as do recent reports of microsporidia being the earliest diverging clade of sequenced fungi (76). A more plausible scenario is that their truncated sequences (e.g., ∼190 bp for V4 region versus ∼252 bp for most bacteria) are removed during length-based filtering steps implemented in most current pipelines (27, 28). Altogether, these findings suggest that reform to certain procedural steps during microbiota data analysis might yield additional information valuable to bee researchers, but should be further validated given the high level of intragenomic variability of rRNA genes in microsporidia (77).

10.1128/mSystems.00082-21.7 FIG S7 *Nosema* reference sequence supplementation does not impact training set classification performance. (A) Segmented regions of the truncated 16S rRNA gene from four *Nosema* spp. aligned to the E. coli K-12 reference 16S rRNA gene showing primer site compatibility for primer sets listed in Data Set S1D. (B) Schematic showing the *Nosema* references that were tested. (C) Classification performance of *BEEx-V4-TS+N* (containing the *Nosema* references) compared with the original *BEEx-V4-TS* using an eight-data set subsample from *nrQS-V4*. *Nosema* ASVs present in the query data set were not included in calculations so as to not inflate classification rates. Data are depicted as the median (line in box), interquartile range (IQR) (box), and minimum/maximum (whiskers) classification rates at each taxonomic rank using ASVs derived from *n* = 8 previously published data sets in *nrQS-V4*. Statistical analysis shown for two-way ANOVA with Tukey’s multiple comparisons. ns, not significant. (D) Prevalence and relative abundance of *Nosema* ASVs. Each point represents the abundance of a *Nosema* ASV from one of the eight subset data sets evaluated. (E) *BEEx-V4-TS* and (F) *BEEx-V4-TS+N* genus- and species-level classification of the unique *Nosema* ASVs. Reference similarity shown on right is in relation to the closest matching sequence representative in the *BEEx-V4-TS+N* training set. Download FIG S7, PDF file, 0.2 MB. Copyright © 2021 Daisley and Reid. 2021 Daisley and Reid. https://creativecommons.org/licenses/by/4.0/ This content is distributed under the terms of the Creative Commons Attribution 4.0 International license.

10.1128/mSystems.00082-21.8 FIG S8 Maximum likelihood phylogeny of V4 ASVs matching *Nosema* spp. relative to mitochondrial and chloroplastic contaminant sequences. Nine bacterial species representing core microbiota members of corbiculate bees are included as points of reference. Bootstrap support values for maximum likelihood greater than 50% are shown on branches. The scale bar represents the number of substitutions per nucleotide site. Download FIG S8, PDF file, 0.2 MB. Copyright © 2021 Daisley and Reid. 2021 Daisley and Reid. https://creativecommons.org/licenses/by/4.0/ This content is distributed under the terms of the Creative Commons Attribution 4.0 International license.

In testing classifiers, our findings support previous comparisons (78) by showing that most commonly implemented algorithms demonstrated similar performances and produced optimal error rates of ∼5% or less during *in silico* testing on simulated error-prone query sequences and ∼18% during cross-validation tests on forced-novel query sequences (Fig. 3A and B). A notable exception, however, was IDTAXA, which exhibited rapid error rate reduction at low-confidence thresholds, rarely made overclassification errors, and displayed approximately fourfold-lower optimal error rates (∼1%) relative to QIIME2-NB and DADA2-NBC classifiers (Fig. S2 and S3). Using IDTAXA with the leading best existing taxonomy reference database (SILVA v138)-derived training sets produced error rates of ∼10% when classifying the same set of simulated bee-derived sequences (Fig. 4A to C), which is slightly lower, but comparable with the ∼17% annotation error estimates of SILVA v128 (16). Importantly, the maximum attainable accurate classification using any of the existing databases did not exceed ∼30%, together suggesting both a poor representation of bee-associated reference sequence as well as incorrect or outdated taxonomy. Providing a demonstratable example, sequences labeled Parasaccharibacter apium in the HoloBee v2016 database are invalid according to recent reports (41) and should be labeled Bombella apis. On a similar note, we draw attention to the fact that the genus *Lactobacillus* was recently overhauled (79), thereby affecting the nomenclature of many bee-associated taxa (e.g., Lactobacillus bombi has been revised to Bombilactobacillus bombi).

We also demonstrated that *in silico* findings could be tightly recapitulated when evaluating previously published 16S rRNA gene sequencing data from 50 different bee hosts across 32 independent studies. Specifically, we report that despite SILVA-based training sets offering nearly identical performance compared to that of BEExact down to the genus level (∼90% or higher), classification rates dropped sharply to ∼28% at the species level (Fig. 5A), which is nearly identical to the *in silico* estimates of ∼30% using the same confidence thresholds (Fig. 4B). In contrast, BEExact enabled persistently higher classification of ∼80% at the species level across most data sets (Fig. 5), which is expectedly lower than *in silico* estimates, but nonetheless demonstrates the habitat-specify and comprehensiveness of the containing database reference sequences from bee host-associated microbial communities. Moreover, we identified several additional advantages, including increased classifier confidence scores when using BEExact (indicator of accuracy), marked improvement in classification of ASVs derived from bee sample origins besides that of gut tissue (e.g., surface, food, larvae), and the classification of 845 ASVs representing novel species which were identifiable by the phylogenetically consistent placeholder names developed in this study (Table 1 and Fig. 5).

Further demonstrating the benefits of a comprehensive habitat-specific database, BEExact enabled classification of several obligate intracellular bacterial pathogens known to infect honey bee hosts (80) including Arsenophonus triatominarum, Arsenophonus nasoniae, Spiroplasma melliferum, and Spiroplasma apis (Data Set S1G and H). At least one or more of these species were successfully identified in the large majority of studies evaluated and especially in the honey bee host-derived data sets in *nrQS-V4*, while SILVA failed to identify any of these important pathobionts. Highlighting the relevance of these findings, *Arsenophonus* spp. (vectored by Varroa destructor mites [81]) are more abundant in honey bee colonies exhibiting clinical signs of colony collapse disorder (81), and *Spiroplasma* spp. can lead to weakened immunity and fatal septic infections in a species-specific manner as well as cross-infect other pollinating insects (2, 82). The ability of BEExact to accurately classify these intracellular pathogens and differentiate associated ASVs at the species level should help to improve our understanding of their virulence, transmission, and cooccurrence—each of which, despite extensive investigation, remains unclear (83).

The small number of ASVs that remained unclassifiable at the species level were partially classified to the genus or family level by BEExact and mostly included members of the *Enterobacteriaceae* from the *nrQS-V4* data set with ambiguous sequence regions that matched at 100% identity with several different taxa—a finding consistent with past literature showing that certain members of the *Enterobacteriaceae* are difficult to distinguish by standard 16S rRNA gene sequencing methods (84). *Gilliamella* spp. (major microbiota members in eusocial corbiculate bees [52]) were also difficult to distinguish using the V4 region alone, and based on whole-genome phylogenomics (60), they should be grouped with the family *Enterobacteriaceae* (order *Enterobacterales*) rather than their current formal designation within the family *Orbaceae* (order *Orbales*). Similarly, Snodgrassella alvi is suggested to belong to the class *Gammaproteobacteria* rather than class *Betaproteobacteria*. These naming conventions are problematic, since they conflict with the widely accepted BFG (*Betaproteobacteria*-*Firmicutes*-*Gammaproteobacteria*) phylotypes, which were established nearly a decade ago (85) and are still frequently referred to as familiar points of reference (86) despite formal designations now existing. In this regard, we highlight that BEExact sequence taxonomy firmly adheres to the List of Prokaryotic Names with Standing in Nomenclature (LPSN) (12) at the genus and species level. However, SILVA-based naming conventions are adopted at higher ranks for several reasons: (i) to enable consistency for comparison to recent literature (22 out of 32 studies assessed used this database; Table 2); (ii) due to SILVA genus names demonstrating the highest degree of congruency with reference sequence in BEExact, thereby reliably connecting species annotations to higher ranks; and (iii) because names are adapted from valid sources (12, 59) as well as curated for maximum phylogenetic accuracy.

### Limitations.

The BEExact database is not equipped to classify rarer environmentally derived species which are not commonly found in bee host-associated microbial communities. It also cannot unambiguously distinguish between short-read sequences from species that share 100% sequence identity within the context of a specific hypervariable region (e.g., V4) but that differ in their full 16S rRNA gene. It is arguable, however, that the former limitation could also be considered an advantage in the sense that outlier taxa and contaminants might more easily be detectable through not readily being classified. In any case, the unclassifiable rare ASVs found across the 32 data sets evaluated were commonly at nearly undetectable levels. Thus, these ASVs are expected to have negligible influence on study findings and based on the prevalence and abundance cutoffs which were established, would mostly be filtered from the data set. With regard to the latter concern, ambiguity in sequence identity of ASVs is not as much a taxonomy reference database constraint as it is an inherent property of short-read sequencing technologies, such as Illumina MiSeq and Ion Torrent S5 which are the predominant platforms currently used for profiling bee microbial communities. This issue is partially accounted via the data-driven recommendation of a classifier (IDTAXA) that is highly accurate and can largely mitigate these types of overclassification errors. However, the full-length BEExact database should also prove useful in the future for nonambiguous classification of full-length 16S rRNA gene amplicons as accuracy and affordability improves for long-read sequencing technologies, such as PacBio and Oxford Nanopore.

### Recommendations.

Based on the findings in this study, the following recommendations are made for future short-read 16S rRNA gene sequencing-based honey bee microbiota studies.

i. For optimal species-level resolution and consistency across all bee lineages, the first choice primer for short-read sequencing (e.g. Illumina MiSeq) should be the V3-V4 targeting primers that were tested in this study (Data Set S1D).
ii. Utilize the latest 16S rRNA pipelines (e.g. DADA2, QIIME2) equipped with denoising algorithms to generate high-resolution ASVs and avoid usage of outdated OTU clustering methods.
iii. Classify ASVs using IDTAXA (recommended) or DADA2-RDP/q2-NB classifiers with conservative bootstrap cutoffs to minimize error rates (see Fig. S2 for optimal cutoff of each gene region tested).
iv. Use region-specific BEExact training sets when classifying short-read ASVs and the full-length BEExact data set when classifying long-read ASVs.
v. Assume that ASVs poorly classified by BEExact are either contamination or derived from rare environmental species, unless there is evidence to suggest the contrary.
vi. Provide sequence data and annotated taxonomy of unique study ASVs when publishing data to advance the cumulative availability of bee-associated 16S rRNA genes.

### Conclusions.

Currently available taxonomy databases hinder classification of the bee microbiota due to a lack of sequence representatives for many habitat-specific ASVs, misrepresentation of references (i.e., labeled identities do not match true taxonomy), and overdiversification of poorly resolved reference sequences—all of which reduce classification confidence and lower the rate of species-level taxonomic assignment independent of the classifier algorithm used. BEExact addresses these shortcomings via an all-inclusive compilation of every known bee-associated 16S rRNA gene sequence publicly available by ensuring that each database reference sequence is accurately annotated (with placeholder names given to uncultured microbial dark matter sequences) and by excluding unrelated sequences of high similarity that can inhibit classifier accuracy due to conflicting taxonomy. Utilizing BEExact alongside the approaches outlined above will facilitate standardized classification of bee-associated microbial communities, improve cross-study reproducibility, and help to highlight novel candidate taxa in need of characterization.

## MATERIALS AND METHODS

### BEExact database construction.

To obtain a list of all known bee (order Hymenoptera, superfamily Apoidea, clade Anthophila)-associated 16S rRNA gene sequences, a comprehensive search was performed on the International Nucleotide Sequence Databases (INSD; http://insdc.org) including the NIH genetic sequence database (GenBank; maintained by NCBI), the European Nucleotide Archive (ENA; maintained by EMBL-EBI) and the DNA Data Bank of Japan (DDBJ). Using the NCBI search portal to achieve this, the following criteria were used in the search command: “(((((((((((((bee) OR bees) OR Andrenidae) OR Apidae) OR Colletidae) OR Halictidae) OR Megachilidae) OR Melittidae) OR Stenotritidae) AND 16S rRNA) AND bacteria[Primary Organism]) OR archaea[Primary Organism]) AND 1000:2000[Sequence Length]) NOT shotgun.” The clade “Anthophila” as is referred to for bees in this study was not included due to overlapping ambiguity with the moth genus Anthophila. In addition, applicable bee host-associated 16S rRNA gene sequences were also collected from recent literature (34–48). This step resulted in the retrieval of 8,869 total 16S rRNA gene sequences.

After compiling these reference sequences in FASTA format, the redundant starting database was first dereplicated to remove strictly identical sequences with priority given to full-length sequences (7,378) using the *derep_fulllength* command of *vsearch* (v2.14.2). Subsequently, sequences that were identical to the prefix of any longer sequence were considered replicates and removed (6,825) using the *derep_prefix* command of *vsearch* (v.2.14.2). Remaining sequences of poor quality that were less than 1,300 bp (1,876) were extracted using Prinseq (v0.20.4) and then matched against full-size 16S rRNA gene reference repositories, including SILVA v138 (SILVA_138.1_SSURef_tax_silva_trunc.fasta.gz; https://ftp.arb-silva.de, GreenGenes v13.8 [GG] (GG_13_8_99.fasta.gz; ftp://greengenes.microbio.me/greengenes_release, GTDB r95 (bac120_ssu_reps_r95.tar.gz; https://gtdb.ecogenomic.org/downloads, and RDP v18 (RDP_v18_current_Bacteria_unaligned.fa; http://rdp.cme.msu.edu/misc/resources.jsp) to obtain reference sequences of higher quality and length using the *usearch_global* command of *vsearch* (2.14.2) with parameters “--id 0.99 --mid 99.”

A positive match in at least one of the databases was identified for 380 sequences. Nonmatches were removed, and the higher-quality references were then merged with the original sequences that were above the 1,300-bp threshold. The revised sequence set was dereplicated once again using the *derep_fulllength* and *derep_prefix* commands of *vsearch* (v2.14.2) to remove redundancy. Next, the sequences were aligned to the 50,000-character global SILVA alignment for rRNA genes with SINA (v1.2.11), trimmed to positions 1044 to 41790 (aligning to the ungapped Escherichia coli 16S rRNA gene reference 28 to 1391 bp), dereplicated again, and then chimeric sequence detection was performed using the SILVA v138 “gold” 16S reference database (https://mothur.org/wiki/silva_reference_files/) with the *uchime* command of *vsearch* (v1.2.11). Trimming of sequences was necessary to remove overhanging 5′ and 3′ ends that would have interfered in downstream accuracy during percent identity calculations. Taxonomic identifiers (NCBI:txid numbers) were retrieved for each sequence accession using the NCBI’s Batch Entrez service (https://www.ncbi.nlm.nih.gov/sites/batchentrez). Associated lineages were then determined in python using default commands “make-acc-taxid-mapping.py” and “make-lineage-csv.py” with the “nucl_gb.accession2taxid” and “taxdump” mapping files available from the NCBI taxonomy FTP site directory (ftp://ftp.ncbi.nlm.nih.gov/pub/taxonomy). Quality steps were then taken via the removal of aberrant eukaryotic, mitochondrial, chloroplastic, and ambiguous nucleotide-containing sequences. Moreover, sequences that were suspiciously short or long within their V4 region (relative to other sequences from the same genus) were removed on the basis that the V4 region should be highly consistent in length between closely related taxonomic group members (87). To do so, the V4-targeting primers in Table 1 were used to trim sequences to the V4 region using the *pcr.seqs* function of *mothur* (v.1.39.5) (88). The lengths of the trimmed V4 sequences in FASTA format were then measured using the Linux command “awk '/^>/{if (l!=””) print l; print; l = 0; next}{l+=length($0)}END{print l}'”. Any sequence that deviated more than 2 bp in length from the mean of its corresponding genus (or lowest common rank membership if genus identity was not available) was omitted from the data set. Following these steps, the intermediate BEExact database contained 4,518 representative 16S rRNA gene sequences.

### BEExact database curation.

Species-level annotation (based on NCBI:txid) was available for only 1,620 of the retrieved sequences. To improve this, the sequence set was queried against type strain material available from large reference databases (SILVA v138, GG v13.8, GTDB r95, and RDP v18) using the *usearch_global* command of *vsearch* (2.14.2) with parameters “--id 0.987 --maxaccepts 0 --maxrejects 0 --uc.” Herein, we refer to percent identity as the number of (matching columns)/(alignment length − terminal gaps), which is the default definition used in most bioinformatic software, including those used in this study. Subsequently, query sequences were assigned taxonomy at the species level based on database hits if identity matched at >98.7%. Lack of consensus between the databases (i.e., when taxonomic names were not consistent between matches at >98.7%) manual refinement was performed by querying of sequences against official representatives (based on NCBI:txid of the matches) retrieved from the Bacterial Diversity Metadatabase (BacDive; https://bacdive.dsmz.de). Moreover, database hits labeled with “Candidatus” were also cross-checked against the List of Prokaryotic names with Standing in Nomenclature (LPSN; https://lpsn.dsmz.de) to ensure conformity with international guidelines and determine the most up-to-date naming. This enabled species-level annotation of 2,233 additional sequences. The 1,620 sequences that already possessed species-level annotations were also included in these search inquires for consistency, which allowed for correction of hundreds of inaccurate or outdated sequence labels.

For the remaining 665 sequences which could not be validly identified at the species level, annotations were instead applied in descending order from domain to genus based on lowest common rank (LCR) (50). Previously established taxonomic identity boundaries (49) (e.g., phylum = 75%, class = 78.5%, order = 82%, family = 86.5%, genus = 94.5%) were used to determine the LCR for each remaining sequence based on percent identity with corresponding database hits from the last step.

Next, to develop placeholder names and allow for consistent reference to unculturable (or yet to be cultured) organisms, we implemented a novel combination of several established distance-based and phylogenetic approaches. Briefly, sequences lacking species-level annotations were grouped based on sequence similarities using the *cluster_smallmem* command of *usearch* (v11.0.667) with parameters “--usersort and --id ###” where ### was set between 0.750 and 0.987 (based on the described phylum to species thresholds [49]) on sequential command entries. Notably, all sequence representatives were used as input (with type strain references coming first in the sequence list, followed by sequences lacking species-level annotations in descending order based on sequence length), and the “--usersort” command was specified to enable consistent group membership under the likely scenario of additional uncultured sequences being added in the future. Next, *de novo* taxonomic labels were generated using the following format wherein #### refers to a unique identifier distinguishing group member at each taxonomic rank: p_bxid#### (phylum), c_bxid#### (class), o_bxid#### (order), f_bxid#### (family), g_bxid#### (genus), and s_bxid#### (species). A recent study successfully utilized a similar approach to develop software-based automatic placement (AutoTax) of sequences to species-level placeholder names while also retaining original sequence identity (i.e., not clustering) (89).

However, identity-based methods alone may result in sequence group memberships which are not phylogenetically accurate due to unequal differences in mutation rate across the 16S rRNA gene (90). To overcome the limitations of distance-based grouping, we applied a maximum likelihood method alongside use of established phylogenetically aware evolutionary placement software (51). First, the intermediate BEExact database was used to search for close neighbor (CN) type strain sequences in the SILVA SSU r138 database using the SINA (v1.2.11) ACT service with parameter [T] in the strain field. A total of 903 CN type strain sequences were retrieved and added to the building database, with type strain taxonomic designations given preference to identical sequences already present after filtering. These CN type strains were considered authoritative points of reference in the case of discrepancies between two or more closely related sequences and were incorporated for the purposes of improving taxonomic resolution in downstream curation steps. Due to potential errors in taxonomic annotation and the tendency of standard databases to be dominated by human-associated taxa, we ensured that bee-associated type strain sequences (as well as recently proposed strains) remained at top authority via dereplication using the *derep_prefix* command of *vsearch* (v2.14.2) to remove shorter type strain sequences with overlapping redundancy.

The CN type strains were aligned to the 50,000-character global SILVA alignment using SINA (v1.2.11), trimmed from positions 1044 to 41790 for compatibility and accuracy in percent identity calculations, and then merged with the intermediate BEExact database. Manual refinement of taxonomy, guided by the most up-to-date and relevant literature sources (16, 60, 91, 92), was then performed to correct conflicts in classification at various taxonomic ranks: *Actinobacteria* (changed to *Actinobacteriota*), *Bacteroidetes* (changed to *Bacteroidota*), *Paenibacillales*;*Paenibacillaceae*;*Brevibacillus* (changed to *Brevibacillales*;*Brevibacillaceae*;*Brevibacillus*), *Acetobacterales* (changed to *Rhodospirillales*), and *Betaproteobacteria*;*Neisseriales*;*Neisseriaceae*;*Snodgrassella* (changed to *Gammaproteobacteria*;*Burkholderiales*;*Neisseriaceae*;*Snodgrassella*). Subsequently, taxonomically mislabeled sequences were identified by using the established Semi-Automatic Taxonomy Improvement and Validation Algorithm (SATIVA) (51) with the command “sativa.py -s input_seqs.phy -t input_tax.txt -x BAC -T 4 -N 1.” Recommendations for adjustment of taxonomy were complemented by manual validation with maximum likelihood-based phylogenetic inference using the RAxML (93) command line “raxmlHPC-HYBRID -T 48 -f a -s input.phy -n output.tre -N 100 -m GTRGAMMA -p 72915 -k -x 77730.” Taxonomic adjustments suggested by SATIVA were made if unanimously supported by bootstrap values of 50 and were consistent with the RAxML output. After all processing steps, the resultant *BEEx-FL-refs* data set (Data Set S1C) had a phylogenetically coherent set of 3,853 sequences with species labels from formal designations and 665 placeholder names based on maximum likelihood-corrected distance-based groupings. The preprocessed nonredundant accession list containing the original 8,869 sequences (Data Set S1A) and the mapping file (Data Set S1B) to representative identifiers in *BEEx-FL-refs* are provided for traceback purposes.

### *In silico* PCR determination of primer biases.

To develop a better understanding of how primer choice may affect honey bee microbiota characterization, standard primer sets targeting various regions of the 16S rRNA gene (Data Set S1D) were queried against the *BEEx-FL-refs* data set containing honey bee-associated reference sequences. Extraction efficiencies were evaluated by *in silico* PCR using the *pcr.seqs* function of *mothur* (v.1.39.5) (88) with 0 mismatches allowed. Ambiguity of sequence identity in the extracted shorter segments was determined by collapsing identical sequences using the *unique.seqs* function of *mothur* (v.1.39.5) with parameter “format=count.” Shannon’s diversity index was then calculated for the resultant count list, which provided a balanced estimate of how different primer biases affect taxonomic evenness and richness. Analysis of primer biases was performed by examining the “scrap.pcr” output files from *pcr.seqs* for each of the primer sets tested.

### Descriptions of query sequence data sets.

Benchmarks performed on error-free sequence queries derived from an identical database as is being used to classify the queries is expected to result in unrealistically inflated performance rates (1). To enable more realistic testing conditions during experiments, error rates of approximately ∼1% were introduced to the sequence representatives derived from *BEEx-FL-refs* using established Mosla Error Simulator (MESA) software (56). Briefly, the ErrASE synthesis method was chosen with the default sequencing method set for paired-end Illumina MiSeq alongside a standard 30-cycle traditional PCR amplification step and a 12-month sample storage period. Three sets of error-prone sequences were generated in this manner for the full-length query sets (*simQS-FL-i* to *-iii*) as well as each of the V3-V4 (*simQS-FL-i* to *-iii*), V4 (*simQS-FL-i* to *-iii*), V4-V5 (*simQS-FL-i* to *-iii*), and V5-V6 (*simQS-FL-i* to *-iii*) hypervariable regions.

To generate *k *= 10 train sets and test (i.e., query) sets for *k*-fold cross validation (57), the *caret* package (v6.0-86) was used in R (v3.6.0). Briefly, full-length or hypervariable region-trimmed sequences were loaded into R using the “readDNAStringSet” function and then randomly sampled (with replacement) using the “sample” function. Subsequently, the “createFolds” command was used with parameters “k = 10, list=TRUE, returnTrain=FALSE” to create the *k *= 10 train and test sets at all regions of interest (e.g., *kQS-FL-##*, *kQS-V3V4-##*, *kQS-V4V5-##*, *kQS-V5V6-##;* where ## is *k *=* 1* to *10* for each set), which were then appended to FASTA format using the “writeXStringSet” function prior to downstream use with classifiers.

The cross-validation by identity (CVI) train and test sets were generated exactly as described previously (50) using TAXXI benchmark software (https://drive5.com/taxxi/doc/index.html) for each region of interest (e.g., *cviQS-FL-##*, *cviQS-V3V4-##*, *cviQS-V4-##*, *cviQS-V4V5-##*, and *cviQS-V5V6-##*, where ## refers to the pair reference for each test and train set).

### Description of training data sets.

In addition to *BEEx-FL-refs*, the other taxonomic reference databases, including SILVA v138 (510,984 sequences), GG v13.8.99 (203,452 sequences), GTDB r95 (21,965 sequences), HBDB (23) (276 sequences; https://treebase.org/treebase-web/search/study/taxa.html?id=13210), and HoloBee v2016 (58) (687 sequences; https://data.nal.usda.gov/dataset/holobee-database-v20161) required various formatting changes prior to use as training sets with the taxonomic classifiers tested in this study.

For use with the naïve Bayesian RDP classifier implemented in DADA2 (DADA2-NBC), training sets were converted to the required FASTA format via the “makeTaxonomy” workflow as described for custom formatted reference databases (https://benjjneb.github.io/dada2/index.html) prior to classifying sequences using the *assignTaxonomy* function of DADA2 in R. For SINTAX compatibility, the FASTA-formatted reference database files were adjusted to include unique accession identifiers at the start of the header (identifiers for *BEEx-FL-refs* in this case) followed by a “tax=” separator and then a colon delimited taxonomic lineage label based on the required specifications (https://drive5.com/usearch/manual/tax_annot.html) prior to taxonomic classification using the “--sintax” command of *vsearch* (v2.14.2). For the naïve Bayes scikit-learn classifier implemented in QIIME2 (QIIME2-NB), reference sequences and associated reference taxonomy were separately imported as QIIME2 artifacts using the “tools import” command with types as “FeatureData[Sequence]” and “FeatureData[Taxonomy],” respectively, prior to downstream classifier training with the q2-feature-classifier (78). For KRAKEN2, custom databases were created as outlined on the tool’s official wiki page (https://github.com/DerrickWood/kraken2/wiki). Briefly, sequences from each of the databases were first imported as KRAKEN2 database images using the *kraken2-build* command with the “--add-to-library” parameter, followed by the *kraken2-build* command with the “--build --db” parameters set to construct the final training sets used to classify sequences. Finally, for IDTAXA, the FASTA-formatted reference sequences were first imported into R using the “readDNAStringSet” function of the DECIPHER package (26). Subsequently, the FASTA headers were parsed and reassembled to include “Root” prior to a semicolon-delimited phylum-to-species taxonomy string for generating a pretrained classifier file with the DECIPHER “LearnTaxa” function, which was then used to classify reads with the “IDTAXA” function.

In addition to format changes, the reference training sets used in this study were trimmed to match hypervariable regions during testing based on reports (1, 94) that trimming of database references to that of the sequenced region of interest can improve the number of reads which are assigned taxonomy (results confirmed independently in Fig. S1C). This was achieved by using the *extract-reads* function of the *q2-feature-classifier* in QIIME2 with default settings with the primers used indicated in Table 1. For simplicity, each taxonomic reference training set is referred to by their full-length (i.e., *xxxxx-FL-TS*) or V#-trimmed (i.e., *xxxxx-V#-TS*) characteristics without consideration given to differences in classifier formatting semantics, wherein “xxxxx” refers to the specific reference database (e.g., *SILVA-V4-TS*). Moreover, training data sets used for taxonomic classification consist of two sets of data: (i) a set of reference sequences and (ii) a corresponding list file mapping each sequence to a hierarchical taxonomy. Thus, both reference sequences and the specific taxonomy applied to them can influence classification efficiency (94). To allow a fair comparison against the 276 honey bee-associated sequence references contained within HBDB (23), the phylotype-level annotations (which were originally assigned at the family level prior to species-level annotations being available) were replaced by the top BLASTn search hit based on the 16S rRNA gene database on NCBI with the “sequences from type material” option indicated. No adjustments were made to the other custom database, HoloBee, since species-level annotations were already provided.

### Classifier settings and performance calculations.

Classifiers were used with the training sets from each reference database to taxonomically classify query sequences in each of the *in silico*-generated data sets, including the simulated (e.g., *simQS-V3V4i-iii*), *k*-fold cross-validation (e.g., *kQS-V3V4-##*), and CVI (e.g., *cviQS-V3V4-##*) data sets.

Taxonomic classification with the naïve Bayesian RDP classifier algorithm (k-mer size = 8) was achieved using the *assignTaxonomy* function of the *DADA2* package in R with 100 bootstrap iterations for each classification run. The minBoot parameter (default = 50%), which sets the minimum required bootstrapping support to return a given taxonomic classification, was tested in increments of 10 with optimal values ranging from 30 to 70.

Both of the QIIME2 classifiers were applied using the q2-feature-classifier command (QIIME2 version 2020.2). For the q2-αHybrid classifier, the *classify-hybrid-vsearch-sklearn* option was used with all settings left as default with the exception of --p-maxhits which was set to “all” and the prefilter setting which was toggled to --p-no-prefilter. For the q2-NB classifier, the “classify-sklearn” option was used with all default settings and read orientation set to “same.” Confidence ranges (default = 70%), which are synonymous to bootstrapping support was tested in increments of 10 with optimal values ranging from 30 to 80 for both classifiers.

The SINTAX classifier implemented in *vsearch* (v2.14.2) was used with all default settings. Confidence cutoffs were tested at increasing increments of 0.1 (range = 0 to 1.0) with optimal ranges between 20 and 60, which are expected to provide comparable accuracy to bootstrap cutoffs of 20 to 60% using the naïve Bayesian RDP classifier.

KRAKEN2 was installed on a Linux operating system and *kraken2* command with parameters “--use-names --confidence ##” for classification. Confidence cutoffs were tested at increasing increments of 0.1 (range = 0 to 1.0) with optimal rates ranging from 0 to 0.5.

The IDTAXA classifier was applied via the DECIPHER package in R using the function “IdTaxa” with the type option set to “extended,” the strand option set to “top,” and the bootstraps option set to “100.” The threshold option (denoting bootstrap support required to classify a sequence) was tested in increments of 10 with optimal ranges between 10 and 50.

The exact script code used for each classifier algorithm is provided for reproducibility in Data Set S2. Following taxonomy assignments, all “NA” and “unclassified” outputs (depending on classifier formatting) were considered equivalent. The raw classification rate at each taxonomic rank was calculated as the percentage of sequence queries assigned any taxonomic label derived from the associated reference training data set. The misclassification rate (MCR) was calculated at each taxonomic rank as the percentage of query sequences assigned a taxonomic label not matching the taxonomy of the parent reference sequence from which it was originally derived. Query sequence that matched multiple references with different taxonomic labels in the parent database (as a result of sequence ambiguity) were not counted toward misclassification rates unless otherwise specified, as this was a classifier-independent feature of data sets. The underclassification rate (UCR) was calculated at each taxonomic rank as the percentage of query sequences that remained unassigned (i.e., did not receive a taxonomic label) following classification. The overclassification rate (OCR) was calculated using previously described software (50) and represents the percentage of sequences classified at a lower rank than possible with the given training set being used in any given test.

10.1128/mSystems.00082-21.10 DATA SET S2 Code for denoising sequencing files with DADA2, ART command lines for *in silico* Illumina MiSeq simulation, IDTAXA classification code, QIIME2 classification code, DADA2 classification code, SINTAX classification code, and KRAKEN2 classification code. Download Data Set S2, DOCX file, 0.04 MB. Copyright © 2021 Daisley and Reid. 2021 Daisley and Reid. https://creativecommons.org/licenses/by/4.0/ This content is distributed under the terms of the Creative Commons Attribution 4.0 International license.

### Retrieval and processing of 16S rRNA gene sequencing data.

A total of 32 past studies were evaluated (Table 3). Raw FASTQ files were directly downloaded from the SRA of the ENA-EBI directory using a custom bash script and the associated FTP site (ftp://ftp.sra.ebi.ac.uk/vol1/fastq). All data sets were first trimmed of adapters and primer binding regions using the Cutadapt (95) command line “cutadapt -e 0.1 -g F_[*V3V4/V4/V4V5/V5V6*] -G R_ [*V3V4/V4/V4V5/V5V6*] -o SRR_filename_out-R1.fastq.gz -p SRR_filename_out-R2.fastq.gz SRR_filename-R1.fastq.gz SRR_filename-R2.fastq.Gz.” Sequence reads were then processed, aligned, and categorized using the DADA2 (v1.8) pipeline to infer exact amplicon sequence variants (ASVs) from amplicon data (7). Briefly, sequence reads were filtered (reads truncated after a quality score of ≤2 and forward/reverse reads truncated after 170/160 bases, respectively) using optimized parameter settings as recommended for the quality profiles (shown in Fig. S5). Next, sequence reads were dereplicated, denoised, and merged using DADA2 default parameters with pooled sample inference implemented for each study data set. A total of 234,567,560 raw reads were processed across the 32 data sets. Following quality assurance measures described in the DADA2 pipeline (27), ASVs were dereplicated using the *derep_fulllength* command of *vsearch* (v2.14.2) and then grouped into four nonredundant hypervariable region-specific data sets, including *nrQS-V3V4*, *nrQS-V4*, *nrQS-V4V5*, and *nrQS-V5V6* containing 6,847, 12,614, 729, and 3,554 total unique ASVs, respectively.

Following retrieval from the SRA database, all data sets were processed similarly through the DADA2 pipeline resulting in a nonredundant set of 6,847 total V3-V4 region ASVs (*nrQS-V3V4*), 12,614 total V4 region ASVs (*nrQS-V4*), 729 total V4-V5 region ASVs (*nrQS-V4V5*), and 3,554 total V5-V6 region ASVs (*nrQS-V5V6*) before removal of contaminants, including ASVs originating from mitochondria, chloroplast, and host bee genomes which were not considered in classification rate calculations (Data Set S1G). To provide guidance in future studies, we first evaluated a single-region subset of ASVs from the largest data set (*nrQS-V4*) to determine how sequence depth impacts the overall quality and comprehensiveness of surveying bee-associated microbial communities. We determined that the total number of detectable ASVs per study was strongly and positively correlated (*R*^2^= 0.9337) with per sample read counts (i.e., read depth; Fig. S4A and B). For outgroup human gut comparisons in Fig. 5C, preprocessed ASV tables from the American Gut Project were downloaded from the ftp site (ftp://ftp.microbio.me/AmericanGut).

### FASTQ generation using MiSeq simulator.

To determine how sequencing depth impacts the resolution of downstream microbial community analysis, several simulated tests at different sampling depths were performed *in silico*. To emulate MiSeq platform-specific sequencing error rates, ART software (62) (Illumina Q version 2.5.8) was implemented in paired-end read simulator mode with customized error profiles modeled based on the hybridized error rates from a subset of eight V4 16S rRNA gene sequencing data sets (Data Set S1G). Briefly, error profiles were first calculated using the ART command line “art_profiler_illumina <read-quality-profile-output> <folder containing subset of FASTQ representatives from each study> fastq.gz 4.” Subsequently, MiSeq (2 × 250 bp) sequencing runs were simulated at 1- to 400-fold read coverage (proxy of sample read depth) using the ART command line “art_illumina -1 read-quality-profile-output_R1.txt -2 read-quality-profile-output_R2.txt -amp -p -sam -na -i *BEEx-V4-refs*.fa -l 250 -f <1-400> -o FASTQ_output/read_depth < 1-400>.Fastq.” The generated FASTQ files were processed identically to that of the empirical sequencing data using the DADA2 pipeline (27) as previously described.

### Statistical analyses.

All statistical analyses were performed using GraphPad Prism (v8.3.0). Data sets were first tested for normality using either the Shapiro-Wilk test for unique values or the D’Agostino-Pearson test for data with two or more identical values. Normally distributed data were statistically compared with one-way or two-way analyses of variance (ANOVAs) with Tukey’s or Sidak’s multiple comparisons where indicated. Nonparametric data sets were statistically compared using Kruskal-Wallis tests with multiple comparisons corrected using the Benjamini-Hochberg false discovery rate method when appropriate.

### Data availability.

NCBI accession numbers for all 16S rRNA gene sequences obtained from public sources are available in Data Set S1. Raw 16S rRNA gene sequencing data sets evaluated in this study are available from the NCBI Sequence Read Archive (BioProject accession nos. PRJNA554741, PRJNA304949, PRJNA348791, PRJNA382070, PRJNA517228, PRJEB22577, PRJEB25500, PRJEB27239, PRJEB27223, PRJNA610196, PRJNA371284, PRJNA491200, PRJNA432210, PRJNA589199, PRJEB23223, PRJEB23224, PRJNA429464, PRJNA225925, PRJNA483763, PRJNA432211, PRJNA578869, PRJNA309422, PRJNA596093, PRJNA530255, PRJNA529891, PRJEB27718, PRJNA485519, PRJNA436176, PRJNA464035, and PRJNA454884), the Dryad international repository (dryad.33518g8), or the Chinese National Genomics Data Center (BioProject accession no. CRA001462). Figures 4 and 5, Fig. S4 to 8, and Data Set S1G to J are associated with this raw data. All other remaining relevant source data are provided in the article, supplemental material, or available from the corresponding author upon request.

## Supplementary Material

Reviewer comments
