# Supplementary figures and images for: BEExact: a Metataxonomic Database Tool for High-Resolution Inference of Bee-Associated Microbial Communities
Source: mSystems. 2021 Apr 6;6(2):e00082-21. doi: 10.1128/mSystems.00082-21 (PMC8546966; doi:10.1128/mSystems.00082-21)

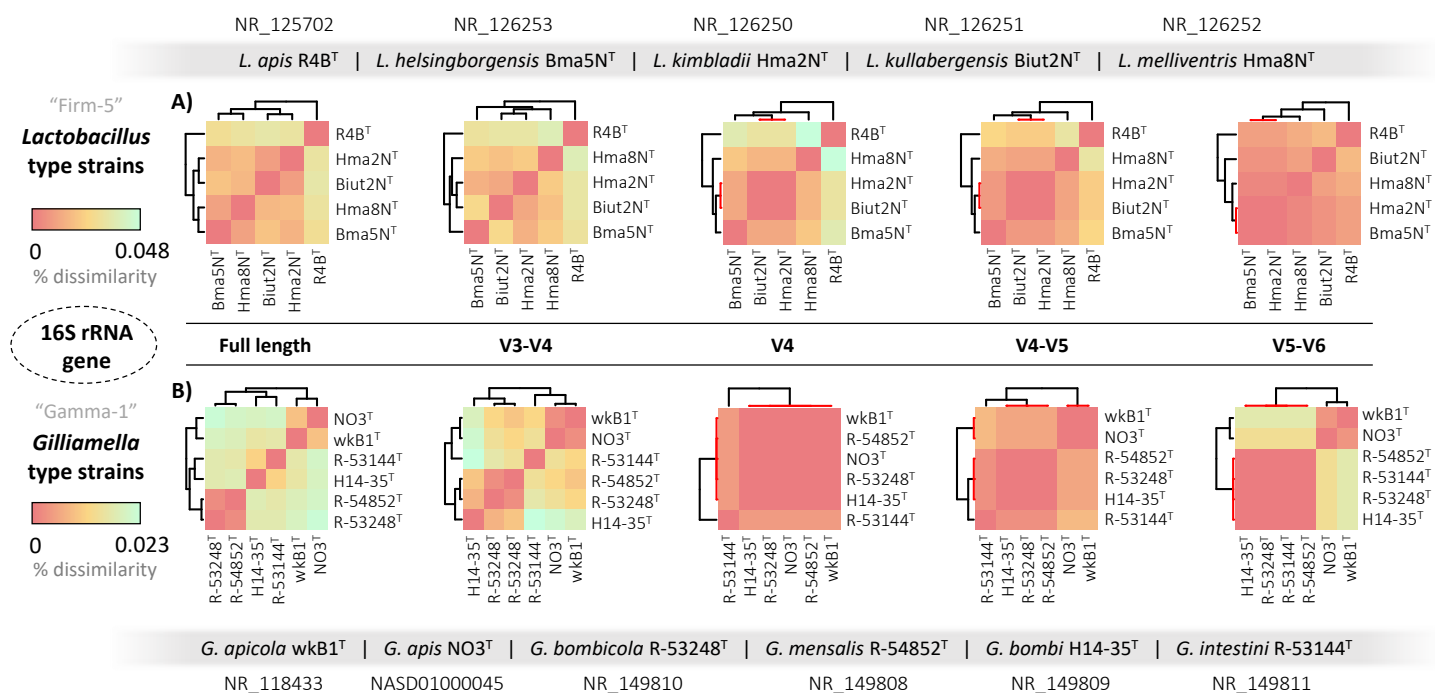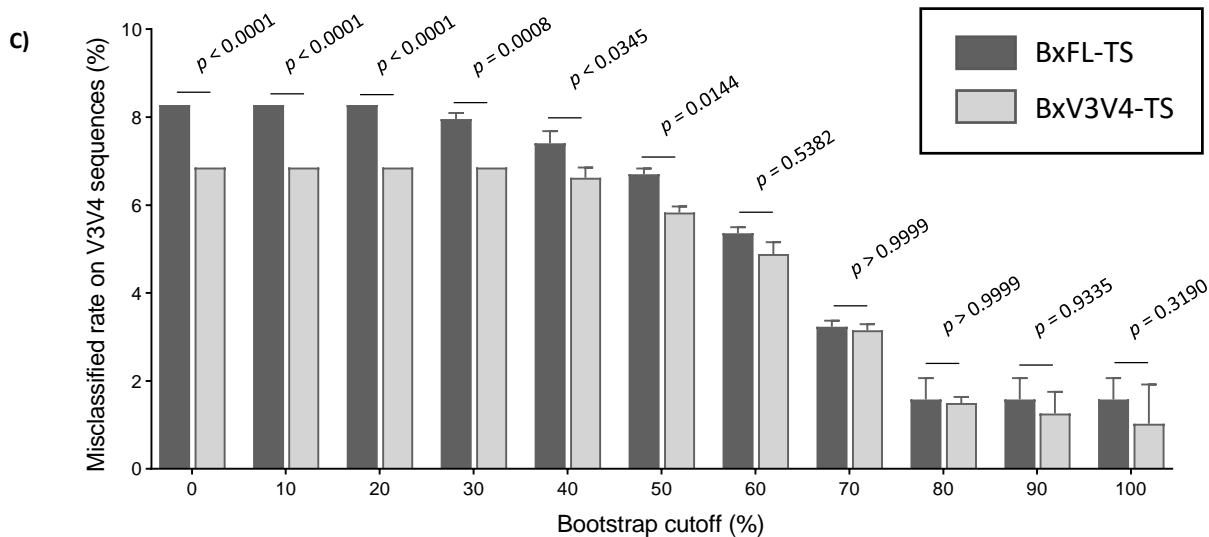

Supplement: FIG S1 [file msystems.00082-21-sf001.pdf]

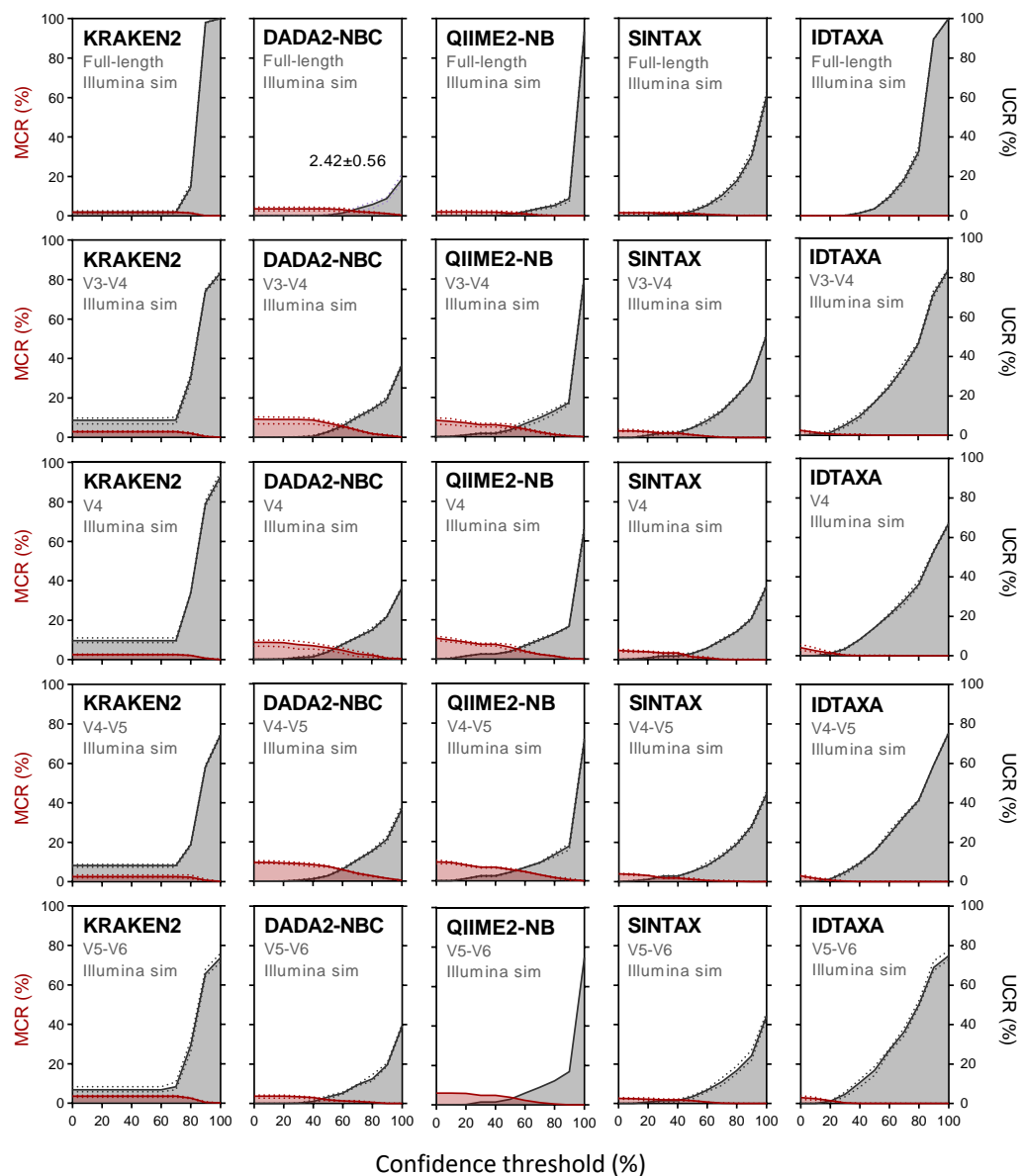

Supplement: FIG S2 [file msystems.00082-21-sf002.pdf]

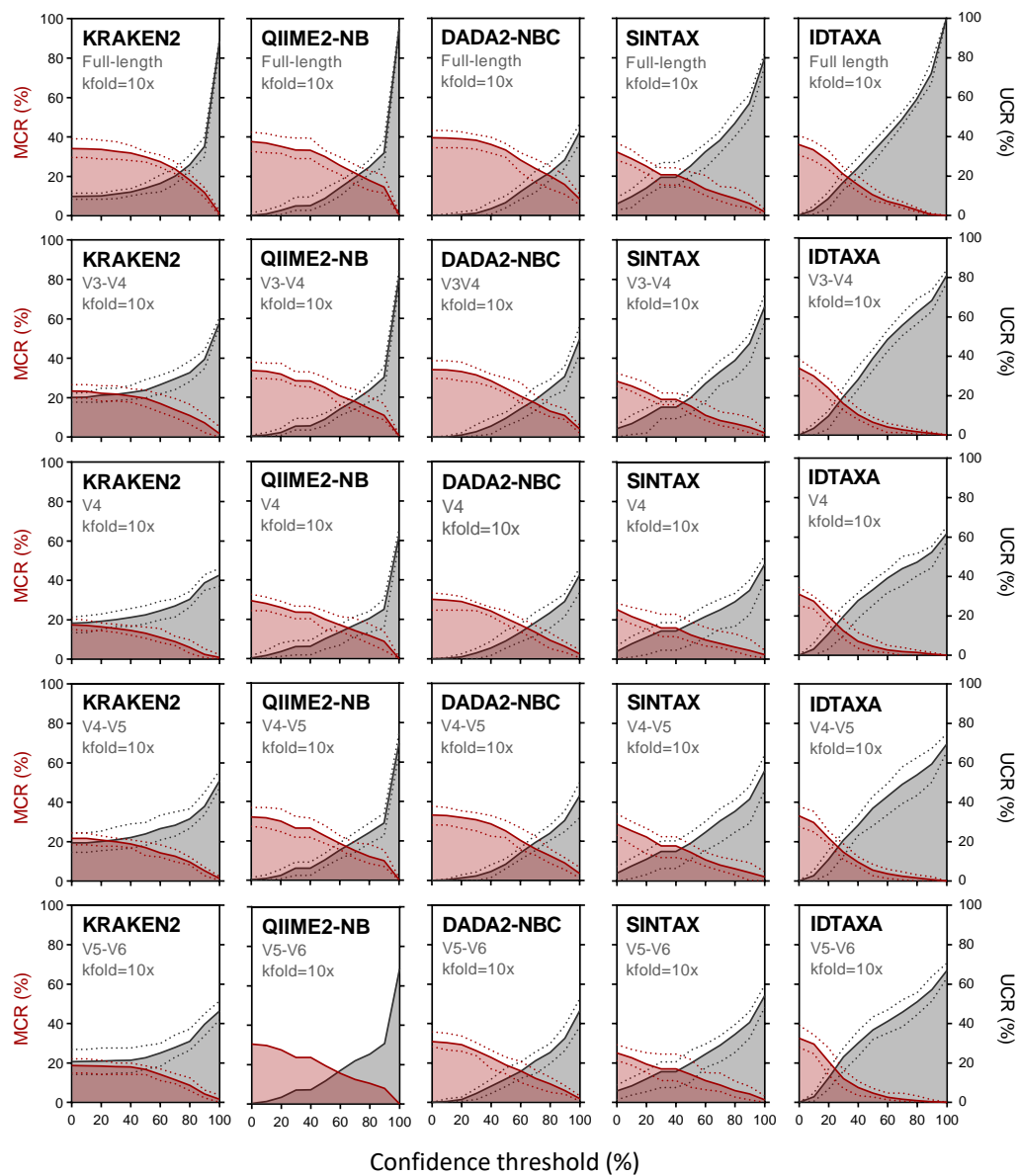

Supplement: FIG S3 [file msystems.00082-21-sf003.pdf]

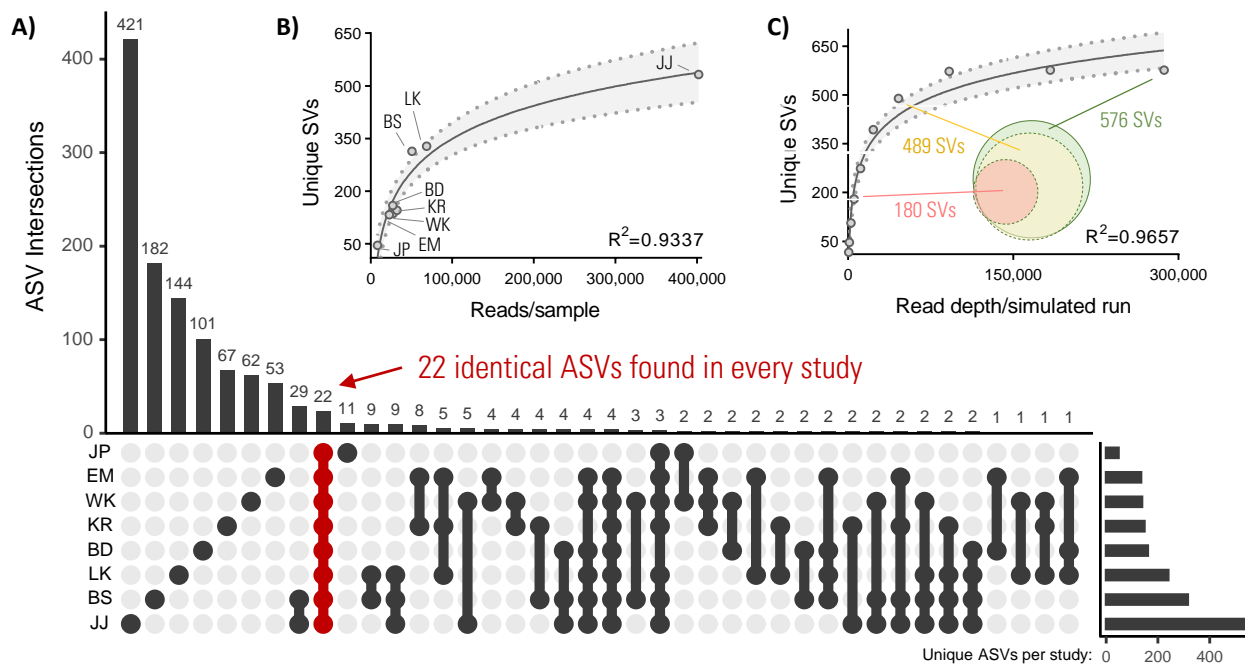

Supplement: FIG S4 [file msystems.00082-21-sf004.pdf]

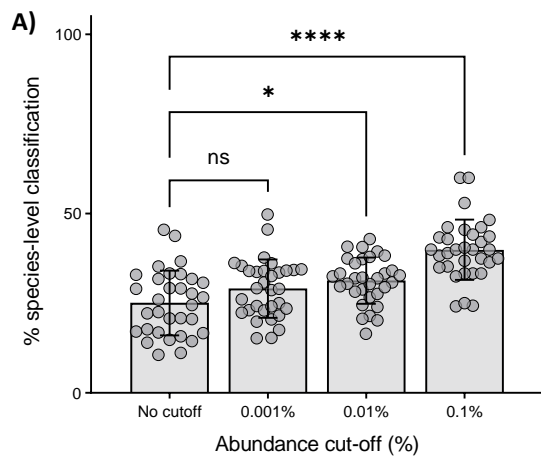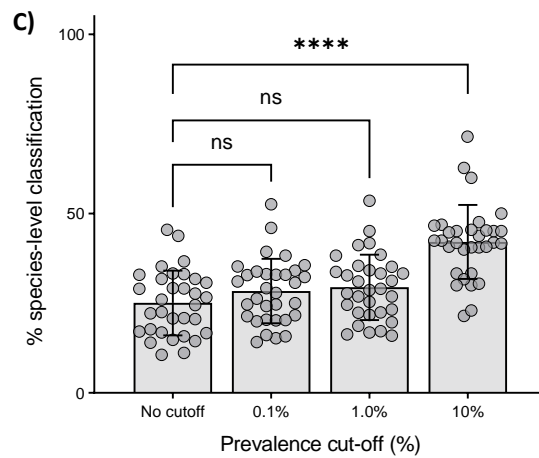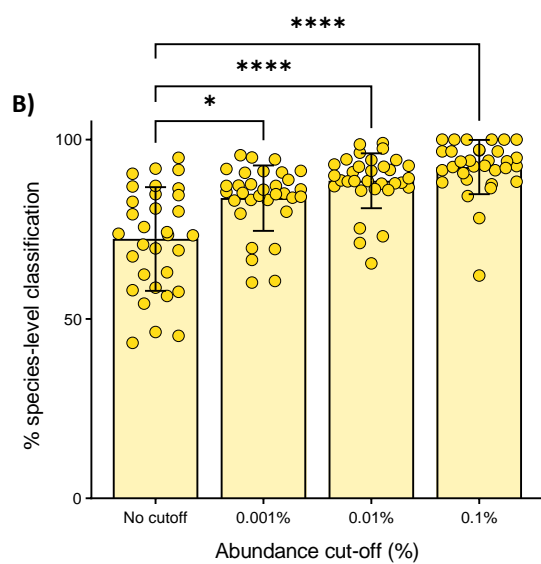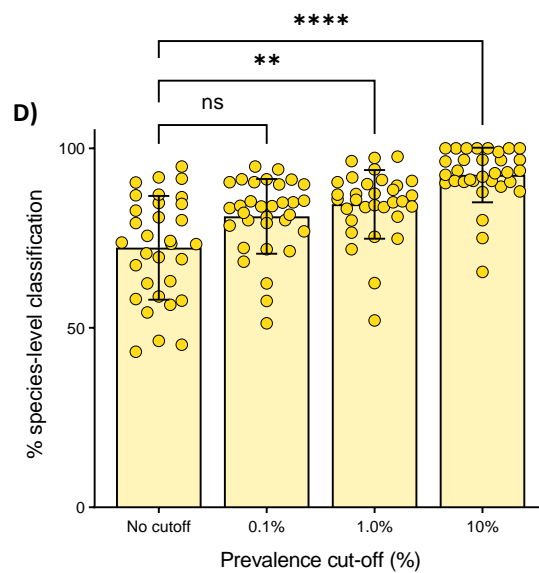

Supplement: FIG S6 [file msystems.00082-21-sf006.pdf]

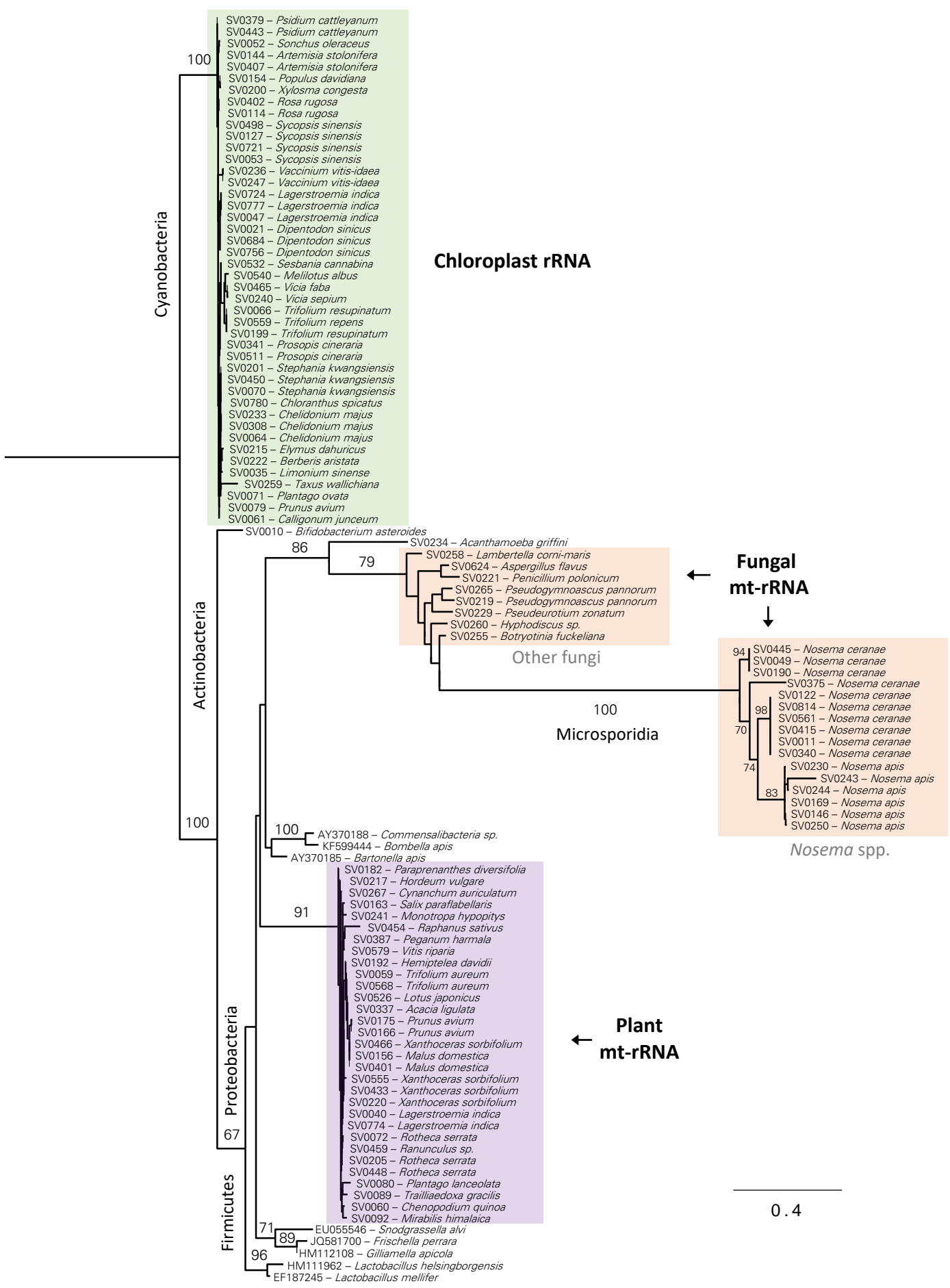

0.4

Supplement: FIG S8 [file msystems.00082-21-sf008.pdf]
