# Supplementary material for: BEExact: a Metataxonomic Database Tool for High-Resolution Inference of Bee-Associated Microbial Communities
Source: mSystems. 2021 Apr 6;6(2):e00082-21. doi: 10.1128/mSystems.00082-21 (PMC8546966; doi:10.1128/mSystems.00082-21)
Supplement: FIG S5 [file msystems.00082-21-sf005.pdf]

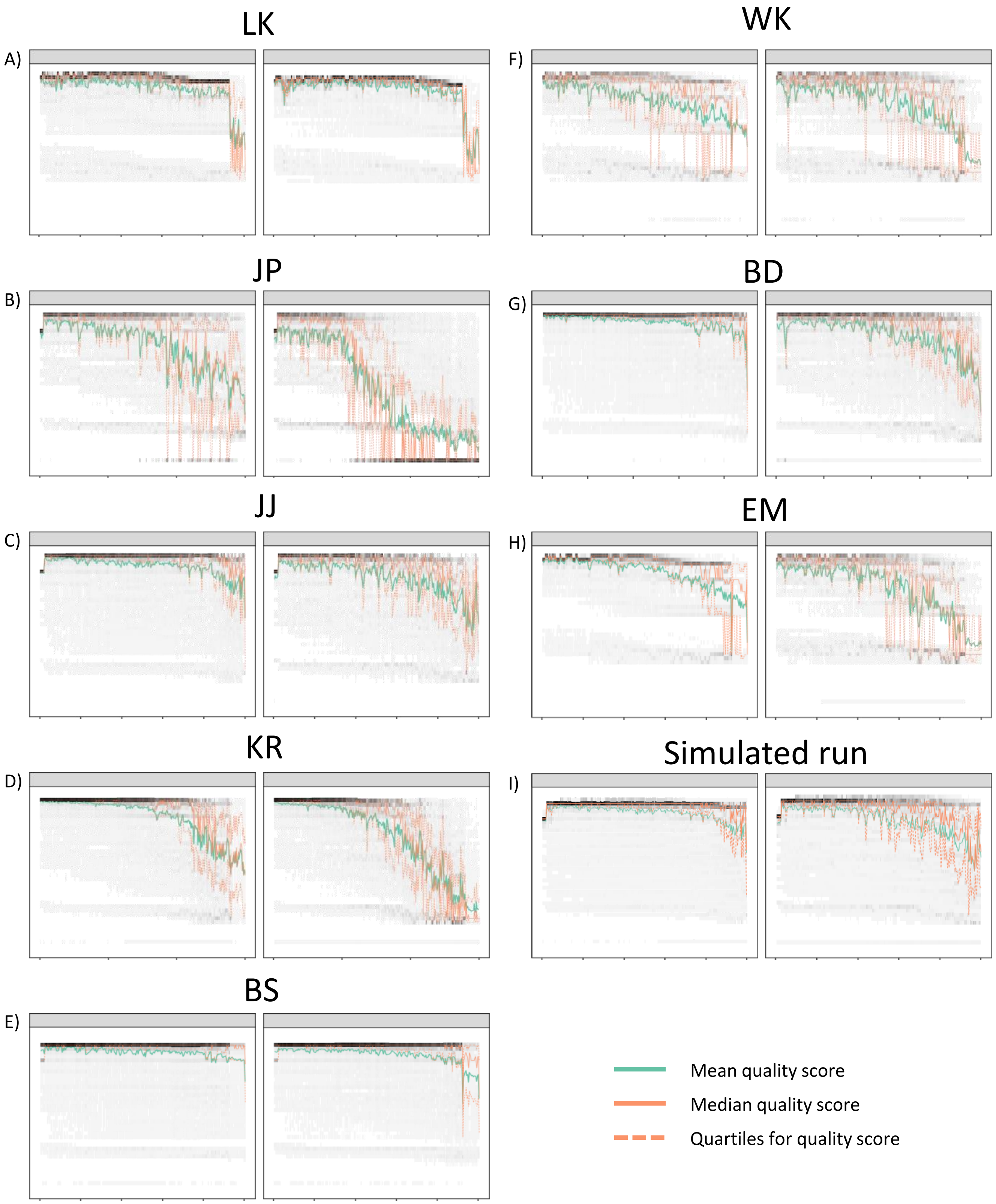
LK
A)
WK
F)
JP
B)
BD
G)
JJ
C)
EM
H)
KR
D)
Simulated run
I)
BS
E)
Mean quality score
Median quality score
Quartiles for quality score
