# Supplementary material for: BEExact: a Metataxonomic Database Tool for High-Resolution Inference of Bee-Associated Microbial Communities
Source: mSystems. 2021 Apr 6;6(2):e00082-21. doi: 10.1128/mSystems.00082-21 (PMC8546966; doi:10.1128/mSystems.00082-21)
Supplement: FIG S7 [file msystems.00082-21-sf007.pdf]

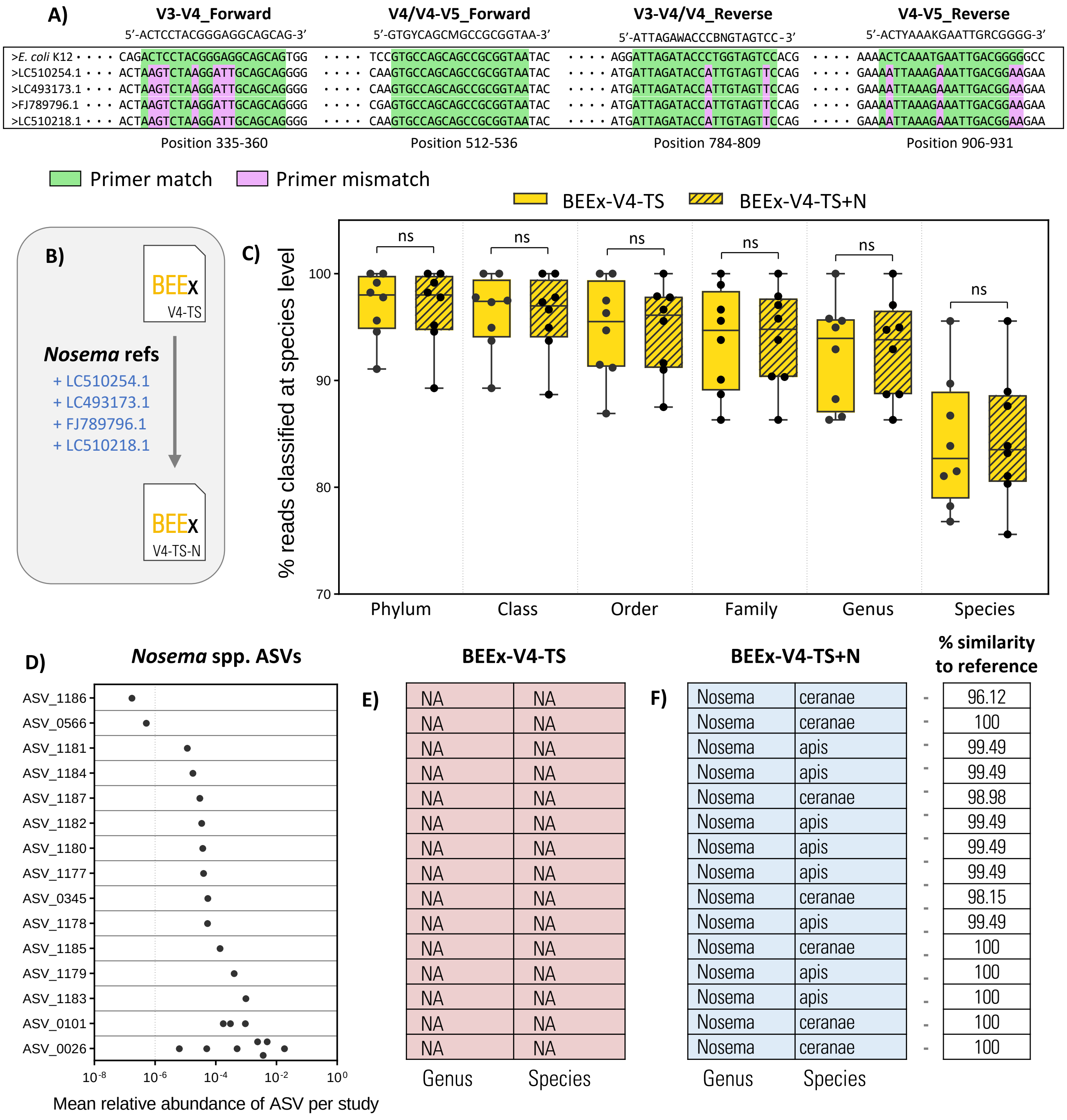

A)
V3-V4_Forward
5'-ACTCCTACGGGAGGCAGCAG-3'
V4/V4-V5_Forward
5'-GTGYCAGCMGCCGCGGTAA-3'
V3-V4/V4_Reverse
5'-ATTAGAWACCCBNGTAGTCC-3'
V4-V5_Reverse
5'-ACTYAAAKGAATTGRCGGGG-3'
>E. coli K12
>LC510254.1
>LC493173.1
>FJ789796.1
>LC510218.1
Position 335-360
Position 512-536
Position 784-809
Position 906-931
Primer match
Primer mismatch
B)
BEEx
V4-TS
Nosema refs
+ LC510254.1
+ LC493173.1
+ FJ789796.1
+ LC510218.1
BEEx
V4-TS-N
C)
BEEx-V4-TS
BEEx-V4-TS+N
% reads classified at species level
ns
Phylum
Class
Order
Family
Genus
Species
D)
Nosema spp. ASVs
ASV_1186
ASV_0566
ASV_1181
ASV_1184
ASV_1187
ASV_1182
ASV_1180
ASV_1177
ASV_0345
ASV_1178
ASV_1185
ASV_1179
ASV_1183
ASV_0101
ASV_0026
Mean relative abundance of ASV per study
E)
BEEx-V4-TS
NA
Genus
Species
F)
BEEx-V4-TS+N
Nosema
ceranae
apis
Genus
Species
% similarity to reference
96.12
100
99.49
99.49
98.98
99.49
99.49
99.49
98.15
99.49
100
100
100
100
100
