# Supplementary material for: BEExact: a Metataxonomic Database Tool for High-Resolution Inference of Bee-Associated Microbial Communities
Source: mSystems. 2021 Apr 6;6(2):e00082-21. doi: 10.1128/mSystems.00082-21 (PMC8546966; doi:10.1128/mSystems.00082-21)
Supplement: DATA SET S2 [file msystems.00082-21-sd002.docx]

**Supplementary Code**

This document contains the following code:

1. Denoising sequencing files with DADA2
2. ART command lines for *in silico* Illumina MiSeq simulation
3. IDTAXA classification code
4. QIIME2 classification code
5. DADA2 classification code
6. SINTAX classification code
7. KRAKEN2 classification code

Denoising sequencing files with DADA2

The denoising algorithm in the DADA2 pipeline was used to process previously published bee-associated 16S rRNA gene sequencing datasets downloaded from SRA as well as simulated sequence run files. In both cases, the demultiplexed starting FASTQ files were handled similarly used the following code with DADA2 package in R.

Official DADA2 webpage: <https://benjjneb.github.io/dada2>

#Load required packages

library(dada2)

#Set your file path to where the demultiplexed FASTQ files are located

#Note: ***FASTQ files should be trimmed of any barcodes and/or primers prior to this step***

reads <- “~/DMXreads”

#Provide filename extension expectations and line up forward(R1)/reverse(R2) reads

fnFs <- sort(list.files(reads, pattern="-R1.fastq.gz", full.names=TRUE))

fnRs <- sort(list.files(reads, pattern="-R2.fastq.gz", full.names=TRUE))

# Get sample names only (remove path, and everything after the first "-")

# Makes assumption that filenames have the format “*filename*-R*.fastq.gz”

sample.names <- sapply(strsplit(basename(fnFs), "-R"), `[`, 1)

#Plot quality profiles

ids<-round(runif(4,1,length(sample.names)))

pdf("quality_profiles.pdf")

plotQualityProfile(fnFs[ids])

plotQualityProfile(fnRs[ids])

dev.off()

# The distribution of quality scores at each position is shown as a grey-scale heat map,

# Summary statistics:

#green line = mean

#orange line = median

#dashed orange lines = 25th and 75th quantiles

#Filtering reads according to quality profiles

#Set filenames for the filtered FASTQ files

filtFs <- paste0(reads, "/", sample.names, "-F-filt.fastq.gz")

filtRs <- paste0(reads, "/", sample.names, "-R-filt.fastq.gz")

#Set parameters

out<-filterAndTrim(fnFs, filtFs, fnRs, filtRs,

truncLen=c(170,160), #optimized based on QC readout

truncQ=2, #quality score cut off after </=2 QC score

trimLeft=c(0,0), #removes bases off the 5' end(R1,R2)

maxN=0,

maxEE=c(2,2),

compress=TRUE, verbose=TRUE, multithread=TRUE)

#Learn R1 and R2 error rates

errF <- learnErrors(filtFs, multithread=TRUE, randomize=TRUE)

errR <- learnErrors(filtRs, multithread=TRUE, randomize=TRUE)

#Plot the error rates

pdf("error_profiles.pdf")

plotErrors(errF, nominalQ=TRUE)

plotErrors(errR, nominalQ=TRUE)

dev.off()

#Dereplicate redundant sequences

derepFs <- derepFastq(filtFs, verbose=TRUE)

derepRs <- derepFastq(filtRs, verbose=TRUE)

#Name the derep-class objects by the sample names

names(derepFs) <- sample.names

names(derepRs) <- sample.names

#Inference sample ASVs, merge paired reads, remove chimeras

#Note: ***If computational power allows, use “pool=TRUE” to enable inferencing across all samples***, this setting greatly improves detection of rare ASV variants which helps better characterize the extensive species-/strain-level variation within microbial communities associated with honey bees

dadaFs <- dada(derepFs, err=errF, multithread=TRUE, pool=TRUE)

dadaRs <- dada(derepRs, err=errR, multithread=TRUE, pool=TRUE)

#Overlap forward/reverse reads

mergers <- mergePairs(dadaFs, derepFs, dadaRs, derepRs, verbose=TRUE)

#Generate sequence table (samples by rows)

seqtab <- makeSequenceTable(mergers)

table(nchar(getSequences(seqtab)))

#Remove chimeras

seqtab.nochim <- removeBimeraDenovo(seqtab, method="pooled", verbose=TRUE, multithread=FALSE)

dim(seqtab.nochim)

#Write output as traditional read count table with ASVs(samples as rows)

write.table(seqtab.nochim, file="ASV_counts.txt", sep="\t", col.names=NA, quote=F)

ART command lines for *in silico* MiSeq simulation

Simulations were performed using ART software available from NIH. First, tailored error rates were generated to match what would be expected during 16S rRNA gene sequencing of honey bee-associated microbial communities. Next, simulations were performed at various read depths to determine the impact on the number of unique ASVs detectable during downstream denoising with DADA2.

Step 1:Download and install ART software for your specific operating system here:

<https://www.niehs.nih.gov/research/resources/software/biostatistics/art/index.cfm>

Here, we used command lines and a Linux-based operating system.

#::::::::::::::::::::::::::::::::::::::::::::::::::::::::::::::::::::

# Generating simulated quality profiles

#::::::::::::::::::::::::::::::::::::::::::::::::::::::::::::::::::::

#If you installed using conda make sure you’re in the right environment

#The input reference folder below contains representative FASTQ files from each of the 8 past bee 16S rRNA gene sequencing datasets evaluated

#For paired-end FASTQ files, as we have here, the files in the reference folder must end in “*_1.fastq.gz” or “*_2.fastq.gz for forward and reverse reverses, respectively

#Number at end of command line is setting the number of CPU cores to use -> most current laptops should have 8+

art_profiler_illumina BEExQP reference_folder fastq.gz 8

#::::::::::::::::::::::::::::::::::::::::::::::::::::::::::::::::::::

# Generating simulated FASTQ files

#::::::::::::::::::::::::::::::::::::::::::::::::::::::::::::::::::::

#Example command line for MiSeq 2x250bp paired-end simulation

#“-1” is simulated forward read quality profiles, “-2” is simulated reverse read quality profiles

# “-i” is input sequences to be simulated, i.e. ASV list of taxa expected to be found in association with honey bees

# “-l” is the sequence read length, here we set to 250 to simulate MiSeq 2x250bp runs

# “-f” indicates the read depth, for this example it is set as 1

# “-o” is output for simulated FASTQ files

art_illumina -amp -p -sam -na \

-1 BEExQP_R1.txt \

-2 BEExQP_R2.txt \

-i BEEx-V4-input.fa \

-l 250 \

-f 1 \

-o BEEx-V4-input_sims/BEEx-V4_sim_f_1.fa

#Code lines used for each simulation at different read depths

#Read depth=1

art_illumina -amp -p -sam -na -1 BEExQP_R1.txt -2 BEExQP_R2.txt -i BEEx-V4-input.fa -l 250 -f 1 -o BEEx-V4-input_sims/BEEx-V4_sim_f_1.fa

#Read depth=2

art_illumina -amp -p -sam -na -1 BEExQP_R1.txt -2 BEExQP_R2.txt -i BEEx-V4-input.fa -l 250 -f 2 -o BEEx-V4-input_sims/BEEx-V4_sim_f_2.fa

#Read depth=4

art_illumina -amp -p -sam -na -1 BEExQP_R1.txt -2 BEExQP_R2.txt -i BEEx-V4-input.fa -l 250 -f 4 -o BEEx-V4-input_sims/BEEx-V4_sim_f_4.fa

#Read depth=8

art_illumina -amp -p -sam -na -1 BEExQP_R1.txt -2 BEExQP_R2.txt -i BEEx-V4-input.fa -l 250 -f 8 -o BEEx-V4-input_sims/BEEx-V4_sim_f_8.fa

#Read depth=16

art_illumina -amp -p -sam -na -1 BEExQP_R1.txt -2 BEExQP_R2.txt -i BEEx-V4-input.fa -l 250 -f 16 -o BEEx-V4-input_sims/BEEx-V4_sim_f_16.fa

#Read depth=32

art_illumina -amp -p -sam -na -1 BEExQP_R1.txt -2 BEExQP_R2.txt -i BEEx-V4-input.fa -l 250 -f 32 -o BEEx-V4-input_sims/BEEx-V4_sim_f_32.fa

#Read depth=64

art_illumina -amp -p -sam -na -1 BEExQP_R1.txt -2 BEExQP_R2.txt -i BEEx-V4-input.fa -l 250 -f 64 -o BEEx-V4-input_sims/BEEx-V4_sim_f_64.fa

#Read depth=128

art_illumina -amp -p -sam -na -1 BEExQP_R1.txt -2 BEExQP_R2.txt -i BEEx-V4-input.fa -l 250 -f 128 -o BEEx-V4-input_sims/BEEx-V4_sim_f_128.fa

#Read depth=256

art_illumina -amp -p -sam -na -1 BEExQP_R1.txt -2 BEExQP_R2.txt -i BEEx-V4-input.fa -l 250 -f 256 -o BEEx-V4-input_sims/BEEx-V4_sim_f_256.fa

#Read depth=400

art_illumina -amp -p -sam -na -1 BEExQP_R1.txt -2 BEExQP_R2.txt -i BEEx-V4-input.fa -l 250 -f 400 -o BEEx-V4-input_sims/BEEx-V4_sim_f_400.fa

IDTAXA classification code

IDTAXA is a classifier available within the DECIPHER package in R. The first block of code below is for training the classifier using a FASTA-based reference file. The second block of code is for classifying a FASTA-formatted sequences with semicolon-delimited taxonomic labels in the header (i.e. >Bacteria;Proteobacteria;etc). Note: If you want to classify your ASVs directly from a traditional read count table that is generated from QIIME2/DADA2 pipelines, you will need to do some conversion steps prior to using the ‘IdTaxa’ command.

Official IDTAXA webpage: <http://www2.decipher.codes/>

Step 1: Training the classifier

#Load necessary packages

library(DECIPHER)

library(data.table)

#Input reference FASTA file with semicolon-delimited taxonomy in headers

dna <-NULL

dna <- readDNAStringSet("~/BEEx-V4-TS.fa")

#Parse the headers to obtain the taxonomy in required data structure

s<- NULL

s <- strsplit(names(dna), ";")

kingdom <- sapply(s, `[`, 1)

phylum <- sapply(s, `[`, 2)

class <- sapply(s, `[`, 3)

order <- sapply(s, `[`, 4)

family <- sapply(s, `[`, 5)

genus <- sapply(s, `[`, 6)

species <- sapply(s, `[`, 7)

taxonomy <- paste("Root", kingdom, phylum, class, order, family, genus, species, sep=";")

head(taxonomy)

#Train the IDTAXA classifier

trainingSet <- LearnTaxa(dna, taxonomy)

#Save classifier for later use

save(trainingSet, file = "IDTAXA-classifier_BEEx-V4-TS.RData")

Step 2: Using the classifier

#Assuming necessary packages are already loaded from the last step

#Load the classifier

BEEx-V4TS_trainingset <- get (load("IDTAXA-classifier_BEEx-V4-TS.RData"))

#Classify reads

Query_seqs <- NULL

query_seqs <- readDNAStringSet("SimQS-V4-i.fa")

ids <- NULL

ids <- IdTaxa(query_seqs,

BEEx-V4TS_trainingset,

type="extended",

strand="both", # or "top" if guaranteed same as the training set used

threshold=20, #20 is recommended based on BEExact benchmarking

bootstraps=100,

processors=NULL) # leave NULL to use all available processors

#Pull out classifications for output file

ids_out <- rbindlist(ids, fill=TRUE, idcol=TRUE)

L <- lapply(split(ids_out, ids_out$.id), function(a) a$taxon)

max_length <- max(lengths(L))

ids_table <- do.call(rbind, lapply(L, function(a) a[1:max_length]))

#Write output file

write.table(ids_table, file="SimQS-V4-i_IDTAXA-classifications_BEEx-V4-TS.txt", sep="\t", col.names=NA, quote=F)

QIIME2 classification code (NB/α-Hybrid classifiers)

Both of these classifiers are implemented in QIIME2 using the q2-feature-classifier. The Naïve Bayes (NB) classifier requires training, whereas the α-Hybrid relies on the NB classifier but does not require any additional training steps. Also, prior to classification the taxonomic reference training set and query sequences need to be converted to QIIME2-compatible file types. Note: The steps below are using FASTA-formatted query sequences. If you want to classify ASVs directly from a count table, you will have to convert file types first. Consider performing prior 16S analysis pipeline steps in QIIME2 to avoid having to do this. For the purposes of the results in this study, no significant differences between these two classifiers and thus only the NB is shown in the results for clarity.

Command lines below are for QIIME2 installed on a Linux-based operating system.

Official QIIME2 webpage: <https://qiime2.org>

#Using QIIME2 (v2020.2)

#:::::::::::::::::::::::::::::::::::::::::::::::::::::::::::

#Importing database files and query sequences

#:::::::::::::::::::::::::::::::::::::::::::::::::::::::::::

#Import full-length reference sequences as “FeatureData[Sequence]” type files

qiime tools import \

--type 'FeatureData[Sequence]' \

--input-path BEEx-FL-TS_q2seqs.fa \

--output-path BEEx-FL-TS_q2seqs.qza

#Import full-length reference taxonomy labels as “FeatureData[Taxonomy]” type files

qiime tools import \

--type 'FeatureData[Taxonomy]' \

--input-format HeaderlessTSVTaxonomyFormat \

--input-path BEEx-q2tax.txt \

--output-path BEEx-q2tax.qza

#Import FASTA-formatted query sequences as “FeatureData[Sequence]” type files

qiime tools import \

--type 'FeatureData[Sequence]' \

--input-path SimQS-V4-i.fa \

--output-path SimQS-V4-i-QS.qza

#Assuming use with V4 primers, extract V4-trimmed reference sequences

qiime feature-classifier extract-reads \

--i-sequences BEEx-FL-TS_q2seqs.qza \

--p-f-primer GTGCCAGCMGCCGCGGTAA \

--p-r-primer GGACTACHVGGGTWTCTAAT \

--p-min-length 100 \

--p-max-length 400 \

--o-reads BEEx-V4-TS_q2seqs.qza

#Training the NB classifier

qiime feature-classifier fit-classifier-naive-bayes \

--i-reference-reads BEEx-V4-TS_q2seqs.qz \

--i-reference-taxonomy BEEx-q2tax.qza \

--o-classifier BEEx-V4-TS_NB-classifier.qza

#:::::::::::::::::::::::::::::::::::::::::::::::::::::::::::

#Example command line for NB classifier

#:::::::::::::::::::::::::::::::::::::::::::::::::::::::::::

qiime feature-classifier classify-sklearn \

--i-classifier BEEx-V4-TS_NB-classifier.qza \

--i-reads SimQS-V4-i-QS.qza

--p-confidence 0.5 \

--o-classification NB-classifications_SimQS-V4-i.qza

#Example command line for visualizing NB classifications

qiime metadata tabulate \

--m-input-file NB-classifications_SimQS-V4-i.qza.qza \

--o-visualization NB-classifications_SimQS-V4-i.qza.qva

# Command lines used for each of the different alpha-Hybrid classification confidence score cut offs

#Confidence=10%

qiime feature-classifier classify-sklearn --i-classifier BEEx-V4-TS_NB-classifier.qza --i-reads SimQS-V4-i-QS.qza --p-confidence 0.1 --o-classification NB-classifications_SimQS-V4-i_10.qza

#Confidence=20%

qiime feature-classifier classify-sklearn --i-classifier BEEx-V4-TS_NB-classifier.qza --i-reads SimQS-V4-i-QS.qza --p-confidence 0.2 --o-classification NB-classifications_SimQS-V4-i_20.qza

#Confidence=30%

qiime feature-classifier classify-sklearn --i-classifier BEEx-V4-TS_NB-classifier.qza --i-reads SimQS-V4-i-QS.qza --p-confidence 0.3 --o-classification NB-classifications_SimQS-V4-i_30.qza

#Confidence=40%

qiime feature-classifier classify-sklearn --i-classifier BEEx-V4-TS_NB-classifier.qza --i-reads SimQS-V4-i-QS.qza --p-confidence 0.4 --o-classification NB-classifications_SimQS-V4-i_40.qza

#Confidence=50%

qiime feature-classifier classify-sklearn --i-classifier BEEx-V4-TS_NB-classifier.qza --i-reads SimQS-V4-i-QS.qza --p-confidence 0.5 --o-classification NB-classifications_SimQS-V4-i_50.qza

#Confidence=60%

qiime feature-classifier classify-sklearn --i-classifier BEEx-V4-TS_NB-classifier.qza --i-reads SimQS-V4-i-QS.qza --p-confidence 0.6 --o-classification NB-classifications_SimQS-V4-i_60.qza

#Confidence=70%

qiime feature-classifier classify-sklearn --i-classifier BEEx-V4-TS_NB-classifier.qza --i-reads SimQS-V4-i-QS.qza --p-confidence 0.7 --o-classification NB-classifications_SimQS-V4-i_70.qza

#Confidence=80%

qiime feature-classifier classify-sklearn --i-classifier BEEx-V4-TS_NB-classifier.qza --i-reads SimQS-V4-i-QS.qza --p-confidence 0.8 --o-classification NB-classifications_SimQS-V4-i_80.qza

#Confidence=90%

qiime feature-classifier classify-sklearn --i-classifier BEEx-V4-TS_NB-classifier.qza --i-reads SimQS-V4-i-QS.qza --p-confidence 0.9 --o-classification NB-classifications_SimQS-V4-i_90.qza

#Confidence=100%

qiime feature-classifier classify-sklearn --i-classifier BEEx-V4-TS_NB-classifier.qza --i-reads SimQS-V4-i-QS.qza --p-confidence 1.0 --o-classification NB-classifications_SimQS-V4-i_100.qza

#:::::::::::::::::::::::::::::::::::::::::::::::::::::::::::

#Example command line for alpha-Hybrid classifier

#:::::::::::::::::::::::::::::::::::::::::::::::::::::::::::

qiime feature-classifier classify-hybrid-vsearch-sklearn \

--i-query SimQS-V4-i-QS.qza \

--i-reference-reads BEEx-V4-TS_q2seqs.qza \

--i-reference-taxonomy BEEx-q2tax.qza \

--i-classifier BEEx-V4-TS_NB-classifier.qza \

--p-strand plus \

--p-confidence 0.5 \

--o-classification aHybrid-classifications_SimQS-V4-i.qza

qiime metadata tabulate \

--m-input-file aHybrid-classifications_SimQS-V4-i.qza \

--o-visualization aHybrid-classifications_SimQS-V4-i.qva

#Command lines used for each of the different alpha-Hybrid classification confidence score cut offs

#Confidence=10%

qiime feature-classifier classify-hybrid-vsearch-sklearn --i-query SimQS-V4-i-QS.qza --i-reference-reads BEEx-V4-TS_q2seqs.qza --i-reference-taxonomy BEEx-q2tax.qza --i-classifier BEEx-V4-TS_NB-classifier.qza --p-strand plus --p-confidence 0.1 --o-classification aHybrid-classifications_SimQS-V4-i_10.qza

#Confidence=20%

qiime feature-classifier classify-hybrid-vsearch-sklearn --i-query SimQS-V4-i-QS.qza --i-reference-reads BEEx-V4-TS_q2seqs.qza --i-reference-taxonomy BEEx-q2tax.qza --i-classifier BEEx-V4-TS_NB-classifier.qza --p-strand plus --p-confidence 0.2 --o-classification aHybrid-classifications_SimQS-V4-i_20.qza

#Confidence=30%

qiime feature-classifier classify-hybrid-vsearch-sklearn --i-query SimQS-V4-i-QS.qza --i-reference-reads BEEx-V4-TS_q2seqs.qza --i-reference-taxonomy BEEx-q2tax.qza --i-classifier BEEx-V4-TS_NB-classifier.qza --p-strand plus --p-confidence 0.3 --o-classification aHybrid-classifications_SimQS-V4-i_30.qza

#Confidence=30%

qiime feature-classifier classify-hybrid-vsearch-sklearn --i-query SimQS-V4-i-QS.qza --i-reference-reads BEEx-V4-TS_q2seqs.qza --i-reference-taxonomy BEEx-q2tax.qza --i-classifier BEEx-V4-TS_NB-classifier.qza --p-strand plus --p-confidence 0.3 --o-classification aHybrid-classifications_SimQS-V4-i_30.qza

#Confidence=40%

qiime feature-classifier classify-hybrid-vsearch-sklearn --i-query SimQS-V4-i-QS.qza --i-reference-reads BEEx-V4-TS_q2seqs.qza --i-reference-taxonomy BEEx-q2tax.qza --i-classifier BEEx-V4-TS_NB-classifier.qza --p-strand plus --p-confidence 0.4 --o-classification aHybrid-classifications_SimQS-V4-i_40.qza

#Confidence=50%

qiime feature-classifier classify-hybrid-vsearch-sklearn --i-query SimQS-V4-i-QS.qza --i-reference-reads BEEx-V4-TS_q2seqs.qza --i-reference-taxonomy BEEx-q2tax.qza --i-classifier BEEx-V4-TS_NB-classifier.qza --p-strand plus --p-confidence 0.5 --o-classification aHybrid-classifications_SimQS-V4-i_50.qza

#Confidence=60%

qiime feature-classifier classify-hybrid-vsearch-sklearn --i-query SimQS-V4-i-QS.qza --i-reference-reads BEEx-V4-TS_q2seqs.qza --i-reference-taxonomy BEEx-q2tax.qza --i-classifier BEEx-V4-TS_NB-classifier.qza --p-strand plus --p-confidence 0.6 --o-classification aHybrid-classifications_SimQS-V4-i_60.qza

#Confidence=70%

qiime feature-classifier classify-hybrid-vsearch-sklearn --i-query SimQS-V4-i-QS.qza --i-reference-reads BEEx-V4-TS_q2seqs.qza --i-reference-taxonomy BEEx-q2tax.qza --i-classifier BEEx-V4-TS_NB-classifier.qza --p-strand plus --p-confidence 0.7 --o-classification aHybrid-classifications_SimQS-V4-i_70.qza

#Confidence=80%

qiime feature-classifier classify-hybrid-vsearch-sklearn --i-query SimQS-V4-i-QS.qza --i-reference-reads BEEx-V4-TS_q2seqs.qza --i-reference-taxonomy BEEx-q2tax.qza --i-classifier BEEx-V4-TS_NB-classifier.qza --p-strand plus --p-confidence 0.8 --o-classification aHybrid-classifications_SimQS-V4-i_80.qza

#Confidence=90%

qiime feature-classifier classify-hybrid-vsearch-sklearn --i-query SimQS-V4-i-QS.qza --i-reference-reads BEEx-V4-TS_q2seqs.qza --i-reference-taxonomy BEEx-q2tax.qza --i-classifier BEEx-V4-TS_NB-classifier.qza --p-strand plus --p-confidence 0.9 --o-classification aHybrid-classifications_SimQS-V4-i_90.qza

#Confidence=100%

qiime feature-classifier classify-hybrid-vsearch-sklearn --i-query SimQS-V4-i-QS.qza --i-reference-reads BEEx-V4-TS_q2seqs.qza --i-reference-taxonomy BEEx-q2tax.qza --i-classifier BEEx-V4-TS_NB-classifier.qza --p-strand plus --p-confidence 1.0 --o-classification aHybrid-classifications_SimQS-V4-i_100.qza

DADA2-RDP classifier

This classifier does not require pre-training, but instead requires a FASTA-formatted input reference training file with headers containing semicolon-delimited taxonomy with no unique headers.

Example header for V4 training set:

>Bacteria;Firmicutes;Bacilli;Bacillales;Bacillaceae;Bacillus;subtilis;

Here, the RDP classifier implemented in the DADA2 package in R was used to classify FASTA-formatted query sequences. Note: Query sequences are being retrieved from their native read count output table format derived from DADA2 pipeline processing of sequencing datasets. If you want to classify ASVs directly from a FASTA files, you will need to do conversion steps prior to using the “assignTaxonomy” command.

Official DADA2 webpage: <https://benjjneb.github.io/dada2>

Official RDP webpage: <https://rdp.cme.msu.edu/classifier>

#Load required packages

library(dada2)

library(vroom)

#Load query ASVs in a count table format

#vroom function used to reduce loading times if dataset is large

seqtab.nochim.vroom <- as.data.frame(vroom("SimQS-V4-i_counts.txt"))

row.names(seqtab.nochim.vroom) <- seqtab.nochim.vroom$samples

seqtab.nochim.vroom$samples <- NULL

seqtab.nochim <- as.matrix(seqtab.nochim.vroom)

#Load classifier

taxpath <- "~/BEEx-V4-TS_DADA2-formatted.fa"

#Example for assigning taxonomy to query ASVs using default settings

taxa <- assignTaxonomy(seqtab.nochim, taxpath, multithread=TRUE, minBoot=50, outputBootstraps = FALSE)

#Reorganize for semicolon-delimited output taxonomy string

#Kingdom to species level is shown here, so 7 levels total, adjust if different

colnames(taxa) <- c("Kingdom", "Phylum", "Class", "Order", "Family", "Genus", "Species")

un.tax <- unname(taxa)

tax.vector <- apply(un.tax, 1, function(x){paste(x[1:7], collapse=";")})

#Genus level

seqtab.nochim.tax<-rbind(seqtab.nochim, tax.vector)

t.seqtab.nochim.tax<-t(seqtab.nochim.tax)

sv.seqs<-rownames(t.seqtab.nochim.tax)

sv.num<-paste("SV", seq(from = 0, to = nrow(t.seqtab.nochim.tax)-1), sep="_")

rownames(t.seqtab.nochim.tax)<-sv.num

#Write output as traditional read count table with taxonomy (samples as rows)

write.table(t.seqtab.nochim.tax, file="DADA2-classifications_SimQS-V4-i.txt", sep="\t", col.names=NA, quote=F)

SINTAX classification

This classifier does not require pre-training, but instead require a FASTA-formatted input reference training file with headers containing colon-delimited taxonomy after a unique sequence identifier (BEExact identifiers in this case but generally accession codes).

Example header:

>BX430200;tax=d:Bacteria,p:Firmicutes,c:Bacilli,o:Bacillales,f:Bacillaceae,g:Bacillus,s:subtilis

Here, the VSEARCH implementation of SINTAX was utilized in a Linux-based operating system to classify FASTA-formatted query sequences. Note: If you want to classify your ASVs directly from a traditional read count table that is generated from QIIME2/DADA2 pipelines, you will need to convert the sequences FASTA prior to using the ‘SINTAX’ command.

Official VSEARCH webpage: <https://github.com/torognes/vsearch>

Official SINTAX webpage: <https://drive5.com/usearch/manual/cmd_sintax.html>

#::::::::::::::::::::::::::::::::::::::::::::::::::::::::::::::::::::

# Classification of ASVs using SINTAX

#::::::::::::::::::::::::::::::::::::::::::::::::::::::::::::::::::::

#Using VSEARCH v2.14.2 implementation of SINTAX

#Example command line for classifying simulated V4 ASV query sequences

vsearch --sintax SimQS-V4-i.fa -db BEEx-V4-TS_SINTAXfomatted.fa \

--sintax_cutoff 0.1 \

--tabbedout SINTAX_SimQS-V4-i-classifications_cutoff10.txt

#Command lines used for each of the different classification confidence score cut offs

#Confidence=10%

vsearch --sintax SimQS-V4-i.fa -db BEEx-V4-TS_SINTAXfomatted.fa --sintax_cutoff 0.1 –tabbedout SINTAX_SimQS-V4-i-classifications_cutoff10.txt

#Confidence=20%

vsearch --sintax SimQS-V4-i.fa -db BEEx-V4-TS_SINTAXfomatted.fa --sintax_cutoff 0.2 –tabbedout SINTAX_SimQS-V4-i-classifications_cutoff20.txt

#Confidence=30%

vsearch --sintax SimQS-V4-i.fa -db BEEx-V4-TS_SINTAXfomatted.fa --sintax_cutoff 0.3 –tabbedout SINTAX_SimQS-V4-i-classifications_cutoff30.txt

#Confidence=40%

vsearch --sintax SimQS-V4-i.fa -db BEEx-V4-TS_SINTAXfomatted.fa --sintax_cutoff 0.4 –tabbedout SINTAX_SimQS-V4-i-classifications_cutoff40.txt

#Confidence=50%

vsearch --sintax SimQS-V4-i.fa -db BEEx-V4-TS_SINTAXfomatted.fa --sintax_cutoff 0.5 –tabbedout SINTAX_SimQS-V4-i-classifications_cutoff50.txt

#Confidence=60%

vsearch --sintax SimQS-V4-i.fa -db BEEx-V4-TS_SINTAXfomatted.fa --sintax_cutoff 0.6 –tabbedout SINTAX_SimQS-V4-i-classifications_cutoff60.txt

#Confidence=70%

vsearch --sintax SimQS-V4-i.fa -db BEEx-V4-TS_SINTAXfomatted.fa --sintax_cutoff 0.7 –tabbedout SINTAX_SimQS-V4-i-classifications_cutoff70.txt

#Confidence=80%

vsearch --sintax SimQS-V4-i.fa -db BEEx-V4-TS_SINTAXfomatted.fa --sintax_cutoff 0.8 –tabbedout SINTAX_SimQS-V4-i-classifications_cutoff80.txt

#Confidence=90%

vsearch --sintax SimQS-V4-i.fa -db BEEx-V4-TS_SINTAXfomatted.fa --sintax_cutoff 0.9 –tabbedout SINTAX_SimQS-V4-i-classifications_cutoff90.txt

#Confidence=100%

vsearch --sintax SimQS-V4-i.fa -db BEEx-V4-TS_SINTAXfomatted.fa --sintax_cutoff 1 –tabbedout SINTAX_SimQS-V4-i-classifications_cutoff100.txt

KRAKEN2 classifier

This classifier requires several steps to be implemented prior to being able to utilize it’s built in classifier function.

In this case, we will assume a FASTA-formatted input reference training file with headers containing UCHIME-format taxonomy.

Example FASTA header:

>BX000342;tax=d:Bacteria,p:Proteobacteria,c:Gammaproteobacteria,o:Burkholderiales,f:Neisseriaceae,g:Snodgrassella,s:Snodgrassella_alvi;

The training set in FASTA format will then be added to a KRAKEN2 library image, followed by building the database to enable classifier usage. To do this, the instructions on the GitHub wiki page for creating a custom KRAKEN2 database were followed. In addition, the Flexible Taxonomy Databases (FlexTaxD) software tool was use to modify files prior to building the KRAKEN2 database.

Official KRAKEN2: <https://ccb.jhu.edu/software/kraken2>

Official KRAKEN2 github wiki: <https://github.com/DerrickWood/kraken2/wiki/Manual>

Official FlexTaxD github page: <https://github.com/FOI-Bioinformatics/flextaxd>

#Assuming a Linux-based operating system with software dependencies already installed

#::::::::::::::::::::::::::::::::::::::::::::::::::::::::::::::::::::

# Generating a KRAKEN2 training set

#::::::::::::::::::::::::::::::::::::::::::::::::::::::::::::::::::::

#Example shows test-train set used with kfold datasets

#Make directory

mkdir kfold_trainsetV3V4_01

#Parse test/train files, expected format in UTAX for this example

cut -d "_" -f 1 ../caret/kfold_trainsetV3V4_01.fasta > \ kfold_trainsetV3V4_01/kracken_kfold_trainsetV3V4_01_cut.fasta

#Replace ">0133" with "bxid0133" ***Make sure command is on 3 lines as shown

sed 's/>/\

>bxid_/g' kfold_trainsetV3V4_01/kracken_kfold_trainsetV3V4_01_cut.fasta > \ kfold_trainsetV3V4_01/kracken_kfold_trainsetV3V4_01_cut_sed.fasta

#Get rid of whitespace at end of lines

sed -i -e 's/[ \t]*//' \ kfold_trainsetV3V4_01/kracken_kfold_trainsetV3V4_01_cut_sed.fasta

sed -i '/^$/d' \ kfold_trainsetV3V4_01/kracken_kfold_trainsetV3V4_01_cut_sed.fasta

#Make trainset names list that is required later

grep "^>" kfold_trainsetV3V4_01/kracken_kfold_trainsetV3V4_01_cut_sed.fasta | awk 'sub(/^>/, "")' > kfold_trainsetV3V4_01/trainsetV3V4_01_nameslist.txt

# Get rid of Windows characters +

# make new training set-specific taxonomy file

grep -w -F -f kfold_trainsetV3V4_01/trainsetV3V4_01_nameslist.txt custom_taxonomy_tabbed_ASCII.txt > kfold_trainsetV3V4_01/trainset01_customtax.txt

#Make custom db <- in "R/kracken2" directory

cd kfold_trainsetV3V4_01

flextaxd --database BEExact_trainsetV3V4_01.db --taxonomy_file trainset01_customtax.txt --taxonomy_type QIIME

#Validate

flextaxd --database BEExact_trainsetV3V4_01.db --validate

#Make nodes.dmp and names.dmp files

flextaxd --database BEExact_trainsetV3V4_01.db --dbprogram kraken2 -o taxonomy --dump

###########################################################################

mkdir seqs_split

cd seqs_split

awk '/^>/{split($1,a,"[>|]")}{print >> a[2]".fna"}' ../kracken_kfold_trainsetV3V4_01_cut_sed.fasta

#Go back up to parent directory

cd ../

#Will say it doesn’t work but keep going

flextaxd-create --database BEExact_trainsetV3V4_01.db -o taxonomy --genomes_path seqs_split --dbprogram kraken2 --create_db --db_name BEExact_trainsetV3V4_01_FINAL --processes 4

rm BEExact_trainsetV3V4_01_FINAL/inspect.txt.gz

###########################################################################

#Pull out just the names part of "seqid2taxid.map" file

awk '{ print $2 > "kraken_names.txt"}' taxonomy/seqid2taxid.map

#Get just the "bxid_####" identifier

cut -d "|" -f 1 kraken_names.txt > kraken_names_bxid.txt

#Combine taxonomy mapping files

paste kraken_names_bxid.txt kraken_names.txt > kraken_names_joined.txt

#Replace names in fasta files by matching with the taxonomy mapping file just created

awk 'FNR==NR{

a[">"$1]=$2;next

}

$1 in a{

sub(/>/,">"a[$1]" ",$1)

}1' kraken_names_joined.txt kracken_kfold_trainsetV3V4_01_cut_sed.fasta > kracken_kfold_trainsetV3V4_01_cut_sed_mapped.fasta

#sed -r -i 's/^(>\S+)\s.*/\1/' kracken_kfold_trainsetV3V4_01_cut_sed_mapped.fasta

sed -r 's/^(>\S+)\s.*/\1/' kracken_kfold_trainsetV3V4_01_cut_sed_mapped.fasta > kracken_kfold_trainsetV3V4_01_cut_sed_mapped1.fasta

mkdir seqs_split_mapped

cp kracken_kfold_trainsetV3V4_01_cut_sed_mapped1.fasta seqs_split_mapped/kracken_kfold_trainsetV3V4_01_cut_sed_mapped1.fna

#cd seqs_split_mapped

#awk '/^>/{split($1,a,"[>|]")}{print >> a[2]".fna"}' ../kracken_kfold_trainsetV3V4_01_cut_sed_mapped1.fasta

#cd ../

############################################################################

find seqs_split_mapped/ -name '*.fna' -print0 | xargs -0 -I{} -n1 kraken2-build --add-to-library {} --db BEExact_trainsetV3V4_01_FINAL --no-masking

cp -R taxonomy BEExact_trainsetV3V4_01_FINAL/taxonomy

rm BEExact_trainsetV3V4_01_FINAL/taxonomy/seqid2taxid.map

#awk '{$1=""; print $0}' BEExact_trainsetV3V4_01_FINAL/taxonomy/seqid2taxid.map > BEExact_trainsetV3V4_01_FINAL/seqid2taxid.map

#awk '{ print $2, $3 }' BEExact_trainsetV3V4_01_FINAL/taxonomy/seqid2taxid.map > BEExact_trainsetV3V4_01_FINAL/seqid2taxid.map

#rm BEExact_trainsetV3V4_01_FINAL/taxonomy/seqid2taxid.map

kraken2-build --build --db BEExact_trainsetV3V4_01_FINAL

cd ../

#Make everything checks out

kraken2-inspect --db BEExact_trainsetV3V4_01_FINAL

#::::::::::::::::::::::::::::::::::::::::::::::::::::::::::::::::::::

# Classification of query datasets using KRAKEN2 built in classifier

#::::::::::::::::::::::::::::::::::::::::::::::::::::::::::::::::::::

#Parse test sets

cut -d "_" -f 1 ../caret/kfold_testsetV3V4_01.fasta > kfold_testsets/kracken_kfold_testsetV3V4_01_cut.fasta

sed 's/>/\

>bxid_/g' kfold_testsets/kracken_kfold_testsetV3V4_01_cut.fasta > kfold_testsets/kracken_kfold_testsetV3V4_01_cut_sed.fasta

sed -i -e 's/[ \t]*//' kfold_testsets/kracken_kfold_testsetV3V4_01_cut_sed.fasta

sed -i '/^$/d' kfold_testsets/kracken_kfold_testsetV3V4_01_cut_sed.fasta

#Classify at each of the confidence thresholds tested

kraken2 --db kfold_trainsetV3V4_01/BEExact_trainsetV3V4_01_FINAL kfold_testsets/kracken_kfold_testsetV3V4_01_cut_sed.fasta > classified/testsetV3V4_01_boots000.txt --use-names --confidence 0

kraken2 --db kfold_trainsetV3V4_01/BEExact_trainsetV3V4_01_FINAL kfold_testsets/kracken_kfold_testsetV3V4_01_cut_sed.fasta > classified/testsetV3V4_01_boots010.txt --use-names --confidence 0.1

kraken2 --db kfold_trainsetV3V4_01/BEExact_trainsetV3V4_01_FINAL kfold_testsets/kracken_kfold_testsetV3V4_01_cut_sed.fasta > classified/testsetV3V4_01_boots020.txt --use-names --confidence 0.2

kraken2 --db kfold_trainsetV3V4_01/BEExact_trainsetV3V4_01_FINAL kfold_testsets/kracken_kfold_testsetV3V4_01_cut_sed.fasta > classified/testsetV3V4_01_boots030.txt --use-names --confidence 0.3

kraken2 --db kfold_trainsetV3V4_01/BEExact_trainsetV3V4_01_FINAL kfold_testsets/kracken_kfold_testsetV3V4_01_cut_sed.fasta > classified/testsetV3V4_01_boots040.txt --use-names --confidence 0.4

kraken2 --db kfold_trainsetV3V4_01/BEExact_trainsetV3V4_01_FINAL kfold_testsets/kracken_kfold_testsetV3V4_01_cut_sed.fasta > classified/testsetV3V4_01_boots050.txt --use-names --confidence 0.5

kraken2 --db kfold_trainsetV3V4_01/BEExact_trainsetV3V4_01_FINAL kfold_testsets/kracken_kfold_testsetV3V4_01_cut_sed.fasta > classified/testsetV3V4_01_boots060.txt --use-names --confidence 0.6

kraken2 --db kfold_trainsetV3V4_01/BEExact_trainsetV3V4_01_FINAL kfold_testsets/kracken_kfold_testsetV3V4_01_cut_sed.fasta > classified/testsetV3V4_01_boots070.txt --use-names --confidence 0.7

kraken2 --db kfold_trainsetV3V4_01/BEExact_trainsetV3V4_01_FINAL kfold_testsets/kracken_kfold_testsetV3V4_01_cut_sed.fasta > classified/testsetV3V4_01_boots080.txt --use-names --confidence 0.8

kraken2 --db kfold_trainsetV3V4_01/BEExact_trainsetV3V4_01_FINAL kfold_testsets/kracken_kfold_testsetV3V4_01_cut_sed.fasta > classified/testsetV3V4_01_boots090.txt --use-names --confidence 0.9

kraken2 --db kfold_trainsetV3V4_01/BEExact_trainsetV3V4_01_FINAL kfold_testsets/kracken_kfold_testsetV3V4_01_cut_sed.fasta > classified/testsetV3V4_01_boots100.txt --use-names --confidence 1.0

#The rest of this code chunk is just getting the readout tables in a human-readable and processable format

awk '{ print $2, $3 }' classified/testsetV3V4_01_boots000.txt > classified/testsetV3V4_01_boots000.txt.taxid

awk '{ print $2, $3 }' classified/testsetV3V4_01_boots010.txt > classified/testsetV3V4_01_boots010.txt.taxid

awk '{ print $2, $3 }' classified/testsetV3V4_01_boots020.txt > classified/testsetV3V4_01_boots020.txt.taxid

awk '{ print $2, $3 }' classified/testsetV3V4_01_boots030.txt > classified/testsetV3V4_01_boots030.txt.taxid

awk '{ print $2, $3 }' classified/testsetV3V4_01_boots040.txt > classified/testsetV3V4_01_boots040.txt.taxid

awk '{ print $2, $3 }' classified/testsetV3V4_01_boots050.txt > classified/testsetV3V4_01_boots050.txt.taxid

awk '{ print $2, $3 }' classified/testsetV3V4_01_boots060.txt > classified/testsetV3V4_01_boots060.txt.taxid

awk '{ print $2, $3 }' classified/testsetV3V4_01_boots070.txt > classified/testsetV3V4_01_boots070.txt.taxid

awk '{ print $2, $3 }' classified/testsetV3V4_01_boots080.txt > classified/testsetV3V4_01_boots080.txt.taxid

awk '{ print $2, $3 }' classified/testsetV3V4_01_boots090.txt > classified/testsetV3V4_01_boots090.txt.taxid

awk '{ print $2, $3 }' classified/testsetV3V4_01_boots100.txt > classified/testsetV3V4_01_boots100.txt.taxid

#Make tabbed query-classification results file

sed -i 's/ / /g' classified/testsetV3V4_01_boots000.txt.taxid

sed -i 's/ / /g' classified/testsetV3V4_01_boots010.txt.taxid

sed -i 's/ / /g' classified/testsetV3V4_01_boots020.txt.taxid

sed -i 's/ / /g' classified/testsetV3V4_01_boots030.txt.taxid

sed -i 's/ / /g' classified/testsetV3V4_01_boots040.txt.taxid

sed -i 's/ / /g' classified/testsetV3V4_01_boots050.txt.taxid

sed -i 's/ / /g' classified/testsetV3V4_01_boots060.txt.taxid

sed -i 's/ / /g' classified/testsetV3V4_01_boots070.txt.taxid

sed -i 's/ / /g' classified/testsetV3V4_01_boots080.txt.taxid

sed -i 's/ / /g' classified/testsetV3V4_01_boots090.txt.taxid

sed -i 's/ / /g' classified/testsetV3V4_01_boots100.txt.taxid

mkdir classified/out

awk 'NR==FNR{tmp[$1]=$2;next};{$2=tmp[$2] ; print }' tax_lineage.match classified/testsetV3V4_01_boots000.txt.taxid > classified/testsetV3V4_01_boots000_out.txt.taxid

awk 'NR==FNR{tmp[$1]=$2;next};{$2=tmp[$2] ; print }' tax_lineage.match classified/testsetV3V4_01_boots010.txt.taxid > classified/testsetV3V4_01_boots010_out.txt.taxid

awk 'NR==FNR{tmp[$1]=$2;next};{$2=tmp[$2] ; print }' tax_lineage.match classified/testsetV3V4_01_boots020.txt.taxid > classified/testsetV3V4_01_boots020_out.txt.taxid

awk 'NR==FNR{tmp[$1]=$2;next};{$2=tmp[$2] ; print }' tax_lineage.match classified/testsetV3V4_01_boots030.txt.taxid > classified/testsetV3V4_01_boots030_out.txt.taxid

awk 'NR==FNR{tmp[$1]=$2;next};{$2=tmp[$2] ; print }' tax_lineage.match classified/testsetV3V4_01_boots040.txt.taxid > classified/testsetV3V4_01_boots040_out.txt.taxid

awk 'NR==FNR{tmp[$1]=$2;next};{$2=tmp[$2] ; print }' tax_lineage.match classified/testsetV3V4_01_boots050.txt.taxid > classified/testsetV3V4_01_boots050_out.txt.taxid

awk 'NR==FNR{tmp[$1]=$2;next};{$2=tmp[$2] ; print }' tax_lineage.match classified/testsetV3V4_01_boots060.txt.taxid > classified/testsetV3V4_01_boots060_out.txt.taxid

awk 'NR==FNR{tmp[$1]=$2;next};{$2=tmp[$2] ; print }' tax_lineage.match classified/testsetV3V4_01_boots070.txt.taxid > classified/testsetV3V4_01_boots070_out.txt.taxid

awk 'NR==FNR{tmp[$1]=$2;next};{$2=tmp[$2] ; print }' tax_lineage.match classified/testsetV3V4_01_boots080.txt.taxid > classified/testsetV3V4_01_boots080_out.txt.taxid

awk 'NR==FNR{tmp[$1]=$2;next};{$2=tmp[$2] ; print }' tax_lineage.match classified/testsetV3V4_01_boots090.txt.taxid > classified/testsetV3V4_01_boots090_out.txt.taxid

awk 'NR==FNR{tmp[$1]=$2;next};{$2=tmp[$2] ; print }' tax_lineage.match classified/testsetV3V4_01_boots100.txt.taxid > classified/testsetV3V4_01_boots100_out.txt.taxid

awk '{ print $1 }' classified/testsetV3V4_01_boots000_out.txt.taxid > classified/join_att1.txt

join -j 1 <(sort classified/testsetV3V4_01_boots000_out.txt.taxid) <(sort classified/testsetV3V4_01_boots000_out.txt.taxid) > classified/join_att2.txt

join -j 1 <(sort classified/join_att2.txt) <(sort classified/testsetV3V4_01_boots010_out.txt.taxid) > classified/join_att3.txt

join -j 1 <(sort classified/join_att3.txt) <(sort classified/testsetV3V4_01_boots020_out.txt.taxid) > classified/join_att4.txt

join -j 1 <(sort classified/join_att4.txt) <(sort classified/testsetV3V4_01_boots030_out.txt.taxid) > classified/join_att5.txt

join -j 1 <(sort classified/join_att5.txt) <(sort classified/testsetV3V4_01_boots040_out.txt.taxid) > classified/join_att6.txt

join -j 1 <(sort classified/join_att6.txt) <(sort classified/testsetV3V4_01_boots050_out.txt.taxid) > classified/join_att7.txt

join -j 1 <(sort classified/join_att7.txt) <(sort classified/testsetV3V4_01_boots060_out.txt.taxid) > classified/join_att8.txt

join -j 1 <(sort classified/join_att8.txt) <(sort classified/testsetV3V4_01_boots070_out.txt.taxid) > classified/join_att9.txt

join -j 1 <(sort classified/join_att9.txt) <(sort classified/testsetV3V4_01_boots080_out.txt.taxid) > classified/join_att10.txt

join -j 1 <(sort classified/join_att10.txt) <(sort classified/testsetV3V4_01_boots090_out.txt.taxid) > classified/join_att11.txt

join -j 1 <(sort classified/join_att11.txt) <(sort classified/testsetV3V4_01_boots100_out.txt.taxid) > classified/join_att12.txt

# Write the final classification table to a tabbed separated file

tr '\r\t' '\t' < classified/join_att12.txt > results/testsetV3V4_01_COMBINED.txt
